# Clinic‐ready inhibitor of MMP‐9/‐12 restores sensory and functional decline in rodent models of spinal cord injury

**DOI:** 10.1002/ctm2.884

**Published:** 2022-05-20

**Authors:** Zubair Ahmed, Sharif Alhajlah, Adam M. Thompson, Rebecca J. Fairclough

**Affiliations:** ^1^ Neuroscience and Ophthalmology Institute of Inflammation and Ageing University of Birmingham Edgbaston UK; ^2^ Centre for Trauma Sciences Research University of Birmingham Edgbaston UK; ^3^ Applied Medical Science College Shaqra University Addawadmi Saudi Arabia; ^4^ Emerging Innovations Unit, Discovery Sciences BioPharmaceuticals R&D AstraZeneca UK

Dear Editor,

This study demonstrated that short‐term inhibition of matrix metalloprotease (MMP)‐9 and MMP‐12 in both mouse and rat models of spinal cord injury (SCI) using the clinic‐ready, orally bioavailable and specific inhibitor, AZD1236, attenuated injury‐induced oedema, proinflammatory pain markers, pain sensation and blood‐spinal cord barrier (BSCB) breakdown. Inhibition of MMP‐9 and MMP‐12 also protected against SCI‐induced sensory and locomotor deficits. By demonstrating these unprecedented improvements with a clinic‐ready MMP inhibitor, using a dosing regimen, which is anticipated to be safe and well tolerated in SCI patients, we are now well‐placed to move swiftly into a Phase 2a study in patients.

After a dorsal column (DC) SCI in mice,^1^ MMP‐9 (Figure 1A) and MMP‐12 (Figure 1B) mRNA peaked at 1 and 5 days, respectively. Protein levels (Figure 1C,D) and enzyme activity (Figure 1E,F) in the spinal cord mirrored their mRNA levels. MMP‐9 and MMP‐12 activity were significantly suppressed in serum and cerebrospinal fluid (CSF) (Figure 1G,H) after oral (Figure 1G,H,K) and intrathecal (Figure 1I–K) delivery of AZD1236, as well as in the spinal cord itself (Figure 1L,M).

**FIGURE 1:**
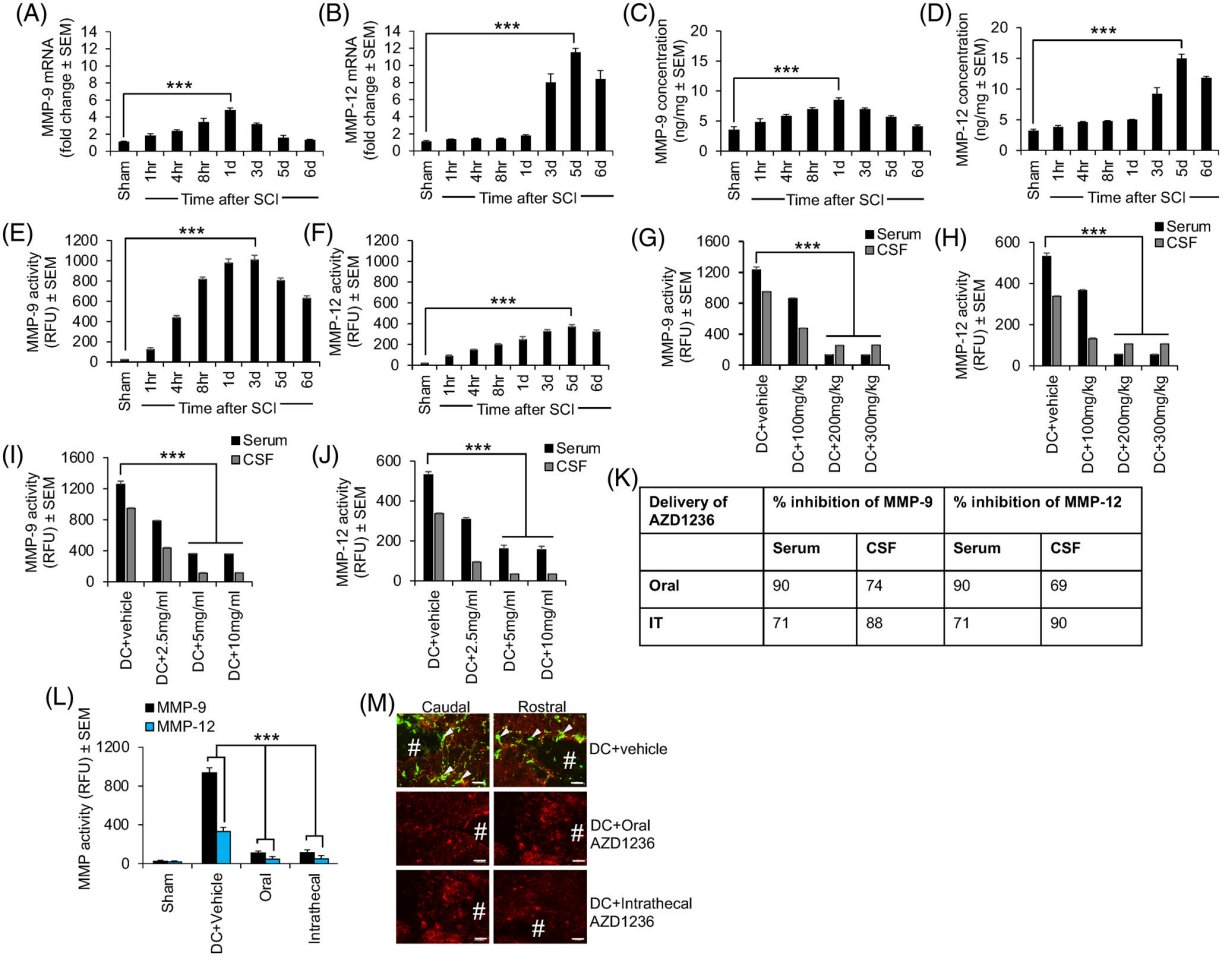
Matrix metalloprotease (MMP)‐9 and MMP‐12 levels and their enzymatic activity increase acutely after dorsal column (DC) injury in mice, and AZD1236 significantly suppresses MMP‐9 and MMP‐12 activity. (A) Levels of MMP‐9 mRNA peak 1 day after injury. (B) Levels of MMP‐12 mRNA peak at 5 days after injury. (C) MMP‐9 protein levels also peak 1 day after injury. (D) MMP‐12 protein levels also peak at 5 days after injury. (E) MMP‐9 activity is high at 1 day and peaks by 3 days after injury. (F) MMP‐12 activity peaks at 5 days after injury. (G) MMP‐9 activity is suppressed by oral delivery of AZD1236 for 3 days after injury in both serum and cerebrospinal fluid (CSF). (H) MMP‐12 activity is also suppressed by oral delivery of AZD1236 in both serum and CSF. (I) MMP‐9 activity is suppressed by intrathecal delivery of AZD1236 for 3 days after injury in both serum and CSF. (J) MMP‐12 activity is also suppressed by intrathecal delivery of AZD1236 in both serum and CSF. (K) Oral delivery of AZD1236 for 3 days after injury suppresses MMP‐9 activity by 90% in both serum and CSF, whilst MMP‐12 is suppressed by 74% and 69% in CSF, respectively. Intrathecal delivery of AZD1236 for 3 days after injury suppresses MMP‐9 activity by 71% in both serum and CSF, whilst MMP‐12 activity is suppressed by 88 and 90% in serum and CSF, respectively. *n* = 6 mice/group. (L) Optimal doses of AZD1236 also significantly suppress MMP‐9 and MMP‐12 activity in spinal cord homogenates at 3 days after injury. RFU = relative fluorescence units. (M) In situ zymography in saggital sections of the lesion site at 3 days after injury shows that the high levels of gelatinase activity (green; arrowheads) after DC injury is suppressed after oral and intrathecal delivery of optimal doses of AZD1236 in spinal cord sections. Sections are counterstained with GFAP (red) to mark astrocytes in red. # = lesion site. Data are expressed as means ± standard error of the mean (SEM). *n* = 6 mice/group, two independent experiments, total *n* = 12 mice/group. *p* = .0001, one‐way analysis of variance (ANOVA) with Dunnett's post hoc test. Scale bars in (M) = 200 μm. NOTE: AZD1236 treatment was provided immediately after injury

After DC injury, water content of the spinal cord peaks at 3 days (Figure S1); however, oral and intrathecal delivery of AZD1236, twice daily for 3 days, caused a dose‐dependent reduction in SCI‐induced water content, with intrathecal delivery requiring much lower doses (5 mg/kg vs. 200 mg/kg) (Figure 2A,B), and inhibition of both MMP‐9 and MMP‐12 was required to fully halt SCI‐induced oedema (Figure 2C). In comparison to pre‐optimised tool and clinical grade experimental MMP inhibitors (Figure S2A–I and Table S1), AZD1236 was significantly more effective at attenuating SCI‐induced water content (Figure S2J).

**FIGURE 2:**
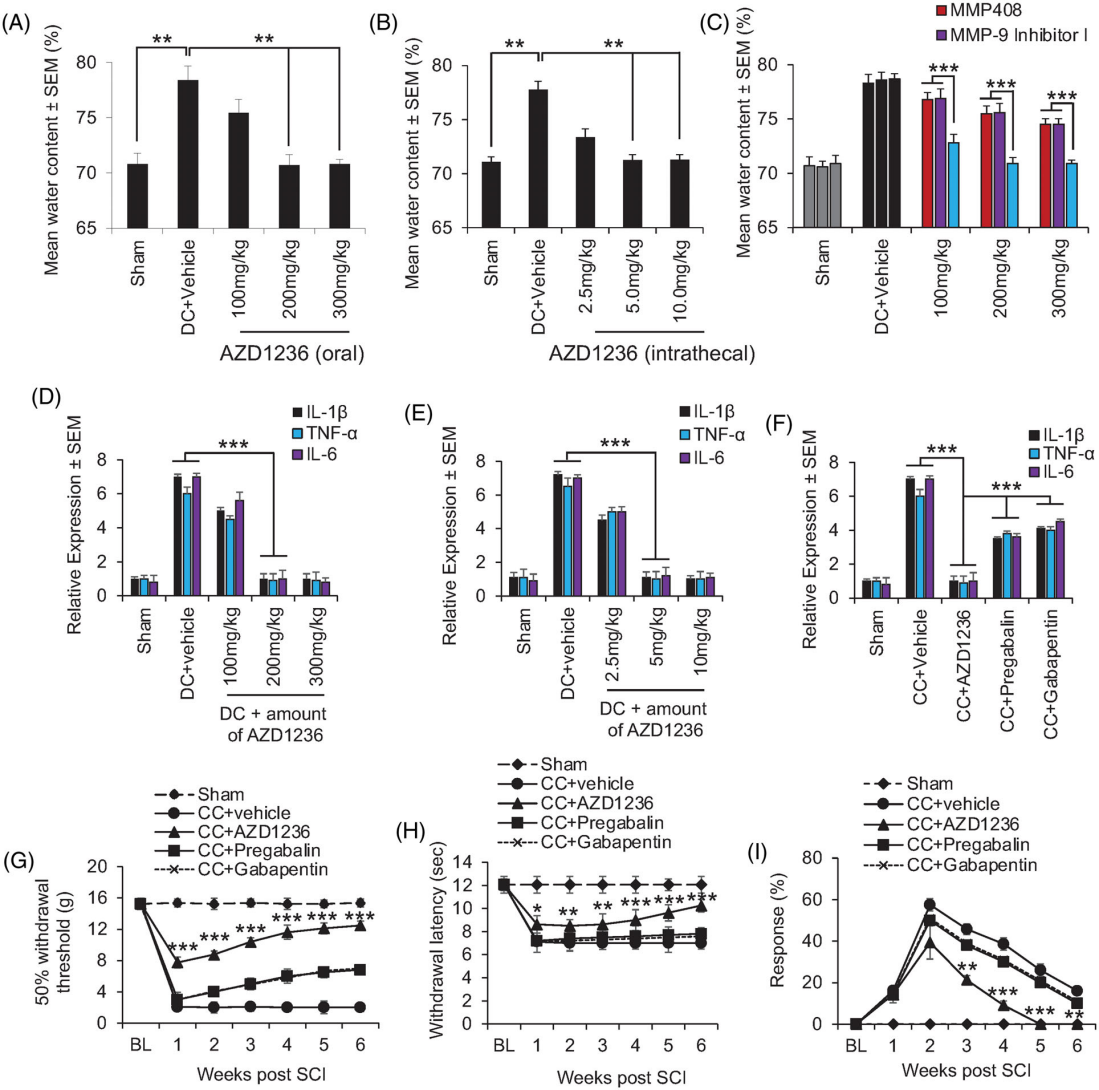
Inhibition of matrix metalloprotease (MMP)‐9 and MMP‐12 using AZD1236 ablates spinal cord injury (SCI)‐induced oedema at 3 days after dorsal column (DC) injury in mice and suppresses proinflammatory pain markers and behavioural correlates of pain. (A) 200 mg/kg of AZD1236 effectively ablated SCI‐induced oedema by oral delivery. (B) 5 mg/kg of AZD1236 effectively ablated SCI‐induced oedema by intrathecal delivery. Data are expressed as means ± SEM. *n *= 6 mice/group, three independent experiments, total *n* = 18 mice/group. (C) Inhibition of both MMP‐9 and MMP‐12 is required to ablate SCI‐induced oedema. Note: MMP408 and Inhibitor I were used at doses above when used singly, however, when combined doses are halved (i.e., 100 mg/kg = 50 mg/kg MMP408/50 mg/kg Inhibitor I, 200 mg/kg = 100 mg/kg MMP408/100 mg/kg Inhibitor I, 300 mg/kg = 150 mg/kg MMP408/150 mg/kg Inhibitor I). Data are expressed as means ± SEM. *n* = 6 mice/group, two independent experiments, total *n* = 12 mice/group. **p = .01; ***p = .0001, one‐way ANOVA with Dunnett's post hoc test. NOTE: AZD1236 treatment was provided immediately after injury. (D) Inhibition of MMP‐9 and MMP‐12 by oral AZD1236 attenuates mRNA levels of proinflammatory pain markers interleukin‐1β (IL‐1β), tumour necrosis factor‐α (TNF‐α) and interleukin‐6 (IL‐6) in the DC injury model. (E) Inhibition of MMP‐9 and MMP‐12 by intrathecal AZD1236 also attenuates mRNA levels of proinflammatory pain markers IL‐1β, TNF‐α and IL‐6 in the DC injury model. (F) Optimal doses of AZD1236 significantly suppress proinflammatory pain markers compared to pregabalin and gabapentin in the DC injury model. (G) Optimal doses of oral AZD1236 significantly improved responses to tactile allodynia over 6 weeks in the clip compression (CC) injury model compared to pre‐optimised doses of pregabalin and gabapentin. (H) Optimal doses of oral AZD1236 significantly improved responses to thermal allodynia over 6 weeks in the CC injury model compared to pre‐optimised doses of pregabalin and gabapentin. (I) Optimal doses of oral AZD1236 significantly improved responses to cold‐induced allodynia over 6 weeks in the CC injury model compared to pre‐optimised doses of pregabalin and gabapentin. Data are expressed as means ± SEM. *n* = 6 mice/group, two independent experiments, total *n* = 12 mice/group. ** = *p* = .01; *** = *p* = .0001, one‐way ANOVA with Dunnett's post hoc test. NOTE: AZD1236 treatment was provided immediately after injury

SCI‐induced pain develops in two thirds of patients,^2^ through increased proinflammatory pain cytokines such as interleukin‐1β (IL‐1β), tumour necrosis factor‐α (TNF‐α) and interkeukin‐6 (IL‐6)^3^ (Figure 2D,E), which were all significantly attenuated by AZD1236 (Figure 2D,E). In a severe clip compression (CC) model of SCI where neuropathic pain develops,^4^ commensurate attenuation of spinal cord water content by AZD1236, (Figure S3A), MMP‐9/MMP‐12 enzyme activity (Figure S3B) and mRNA expression of IL‐1β, TNF‐α, IL‐6 (Figure S3C) to that seen in the DC model was also observed. Interestingly, inhibition of MMP‐9 and MMP‐12 using AZD1236 was superior than pre‐optimized doses of pregabalin and gabapentin at suppressing IL‐1β, TNF‐α and IL‐6 (Figure 2F) and attenuating tactile‐ (Figure 2G), thermal‐ (Figure 2H) and cold‐induced allodynia (Figure 2I).

Treatment with oral AZD1236 significantly suppressed BSCB breakdown (Figure 3A,B), reduced fibrotic scarring (Figure 3C,D), suppressed Semaphorin‐3A (Sema‐3A) (Figure 3E,F) and CS‐56 levels (Figure 3G,H), attenuated CD11b^+^ (Figure 3I,J), CD68^+^ (Figure 3I,K) and glial fibrillary acidic protein (GFAP)^+^ (Figure 3I,L) immunoreactivity at the lesion site. In isolated primary adult mouse brain microglia subjected to lypopolysaccharide (LPS) activation, increasing doses of AZD1236 caused a dose‐dependent decrease in proinflammatory cytokines (Figure S4A–C) compared to other tool MMP inhibitors (Figure S4A–C) but chemotaxis in primary macrophages or macrophage cell lines (J744A.1 and RAW 264.7) was unaffected (Figure S4D).

**FIGURE 3:**
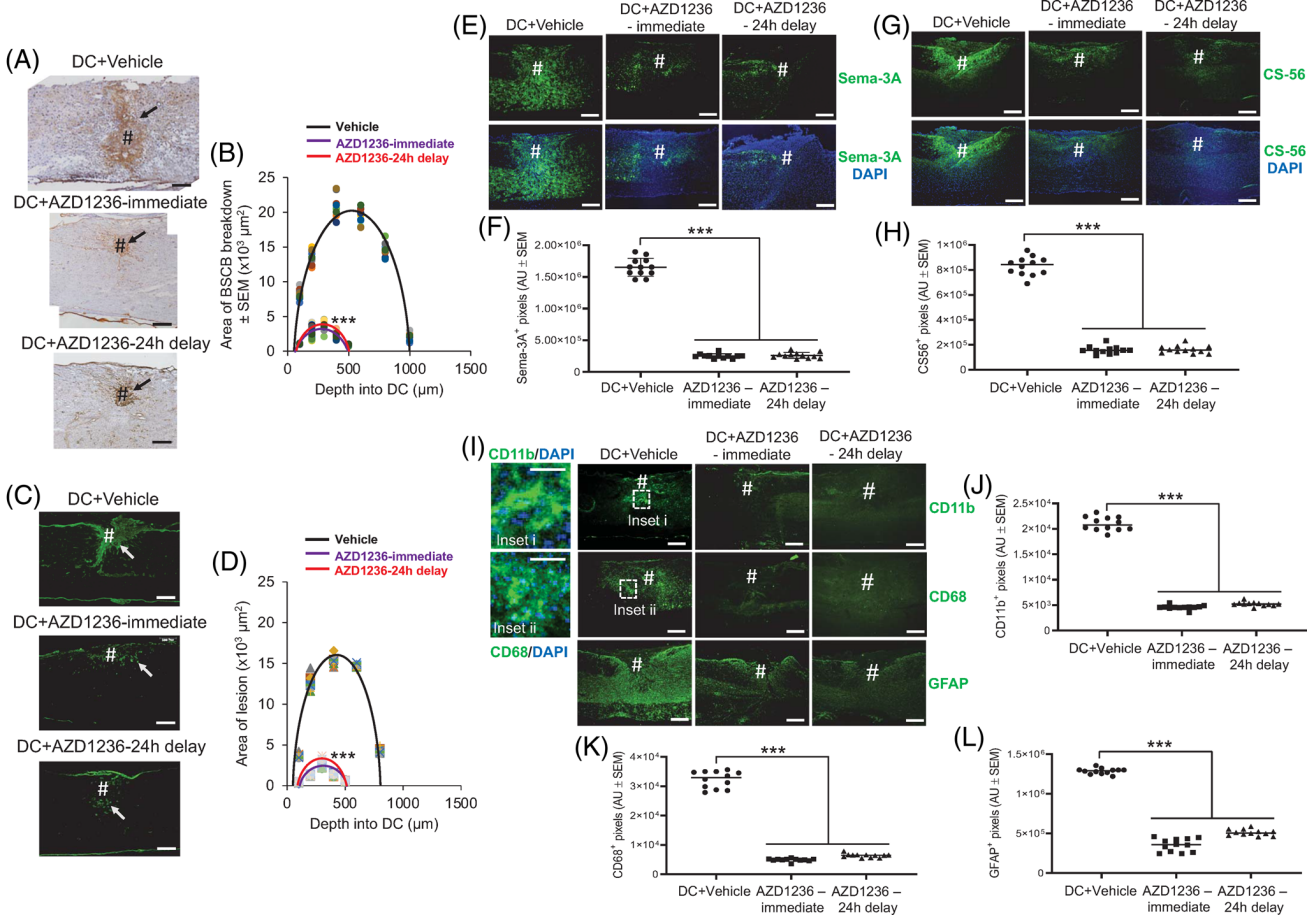
Inhibition of matrix metalloprotease (MMP)‐9 and MMP‐12 attenuates blood spinal cord barrier (BSCB) breakdown and scar tissue at the mouse lesion site. (A) Inhibition of MMP‐9 and MMP‐12 suppressed albumin extravasation as detected by albumin immunoreactivity at lesion site (#) at 3 days after dorsal column (DC) injury and treatment with AZD1236 compared to vehicle‐treated animals. Scale bars = 100 μm. (B) Quantification of the amount of albumin immunoreactivity reflected these changes to demonstrate suppressed BSCB breakdown. Individual data points shown by different colour symbols. (C) Laminin immunoreactivity (scar tissue) at the lesion site (#) at 4 weeks after DC injury and treatment showed significantly lower levels of immunoreactivity in AZD1236‐treated animals compared to vehicle‐treated groups. Scale bars = 100 μm. (D) Quantification of the number of laminin immunoreactive pixels demonstrated significantly attenuated levels of laminin scar tissues at the lesion site. Individual data points shown by different colour symbols. Immunohistochemistry and quantification in saggital sections of the lesion site for Semaphorin‐3A (Sema‐3A) (E and F) and CS‐56 (G and H) at 7 days after DC injury and treatment, respectively. (I and J) CD11b, (I and K) CD68 and (I and L) glial fibrillary acidic protein (GFAP) immunoreactivity and quantification in sagittal sections from DC+vehicle and DC+AZD1236‐treated animals at 10 days after injury, respectively. Scale bars in E, G and I = 100 μm, in inset i and inset ii = 200 μm. (J–L) Quantification of the number of CD11b^+^, CD68^+^ and GFAP^+^ immunoreactive pixels demonstrated significantly suppressed levels in DC+AZD1236 treated animals. Data are expressed as means ± SEM. AU = arbitrary units. *n* = 6 mice/group, two independent experiments, total *n* = 12 mice/group. ***p = .0001, one‐way ANOVA with Dunnett's post hoc test. NOTE: AZD1236 treatment was provided immediately and 24 h after injury, as indicated on figure label

AZD1236 treatment promoted significant axon regeneration (Figure 4A,B) compared to other MMP inhibitors (Figure S5) and increased neurofilament 200 (NF200)^+^ spared fibres in the spinal cord above (T9) and below (T7) the lesion (Figure 4C,D). CSF and serum biomarkers of SCI were also significantly attenuated by AZD1236 (Figure S6A–E). Axon regeneration and enhanced sparing of axons after SCI correlated with improved electrophysiological function (Figure 4E–G) and significant improvements in locomotor (Figure 4H) and sensory function (Figure 4I), with intrathecal delivery of AZD1236 having similar benefits (Figure 4J–N). Moreover, oral and intrathecal AZD1236 treatment in the severe CC injury model at the same lowest effective dose as the DC model, also significantly improved electrophysiological function across the lesion site (Figure S7A–C) and locomotor performance (Figure S7D,E).

**FIGURE 4:**
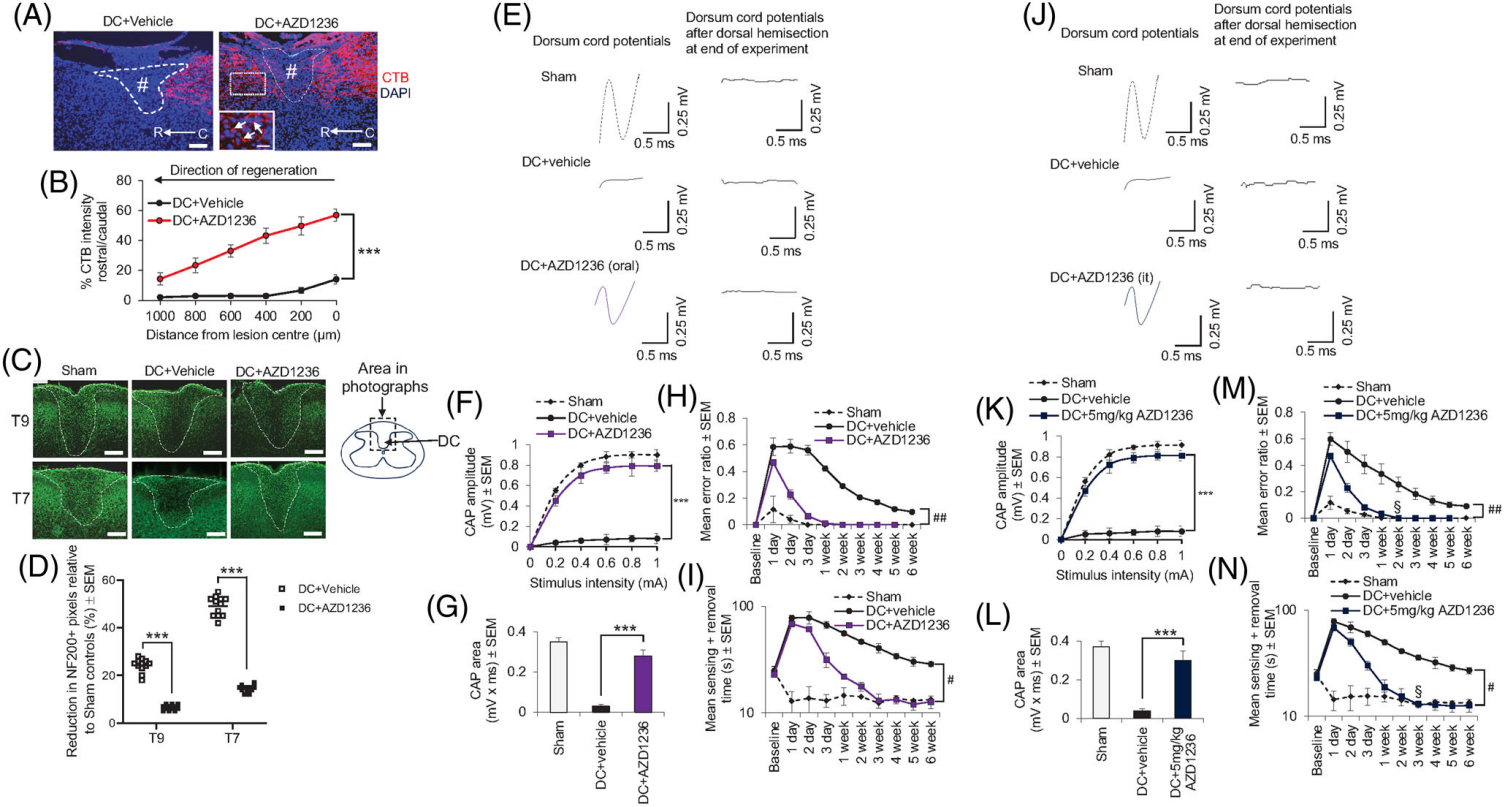
Inhibition of matrix metalloprotease (MMP)‐9 and MMP‐12 using AZD1236 promotes dorsal column (DC) axon regeneration, axon sparing and improves electrophysiological, locomotor and sensory outcomes after DC injury in mice. (A) Cholera toxin B (CTB) retrogradely labelled axons to regenerating/sprouting within the DC lesion site and growing into the rostral cord of animals treated with DC+AZD126, while no evidence of CTB labelled axons was present in the lesion site or beyond in DC+vehicle‐treated controls. Scale bars = 200 μm. (B) Significantly greater proportions of CTB‐labelled axons were quantified rostra/caudal to the lesion site in DC‐AZD1236‐treated animals compared to DC+vehicle‐treated controls. (C) Immunohistochemistry to detect neurofilament (NF)200^+^ fibres in cross sections of the spinal cord from DC+vehicle and DC+AZD1236‐treated animals, above (T9) and below (T7) the lesion site. Scale bars = 200 μm. (D) Quantification of the number of NF200 pixels above and below the lesion site to demonstrate sparing of axons in DC+AZD1236‐treated animals compared to DC+vehicle‐treated controls. Data are expressed as means ± SEM. *n* = 6 mice/group, two independent experiments, total *n* = 12 mice/group. ***p = .0001, one‐way ANOVA with Dunnett's post hoc test. (E) Representative Spike 2 processed compound action potential (CAP) traces after oral delivery of AZD1236 showing ablation of CAP waves at 6 weeks after DC injury but restoration of a significant CAP wave by oral AZD1236. (F) Oral AZD1236 significantly improved CAP amplitudes at 6 weeks after DC injury. (G) Oral AZD1236 significantly improved CAP areas at 6 weeks after DC injury. (H) Oral AZD1236 significantly improved ladder crossing performance (locomotor function) at 6 weeks after DC injury. (I) Oral AZD1236 significantly improved tape sensing and removal performance (sensory function) over 6 weeks after DC injury. (J) Representative Spike 2 processed CAP traces after intrathecal delivery of AZD1236 showing ablation of CAP waves over 6 weeks after DC injury but restoration of a significant CAP wave by intrathecal AZD1236. (K) Intrathecal AZD1236 significantly improved CAP amplitudes at 6 weeks after DC injury. (L) Intrathecal AZD1236 significantly improved CAP areas at 6 weeks after DC injury. (M) Intrathecal AZD1236 significantly improved ladder crossing performance (locomotor function) over 6 weeks after DC injury. (N) Intrathecal AZD1236 significantly improved tape sensing and removal performance (sensory function) over 6 weeks after DC injury. Data are expressed as means ± SEM. *n* = 6 mice/group, three independent experiments, total *n* = 18 mice/group. ***p = .0001, one‐way ANOVA with Dunnett's post hoc test. ^§^ *p* = .0001, independent sample *t*‐test. ^#^ *p* = .0015, linear mixed models; ^##^ *p* = .0011, generalized linear mixed models. NOTE: AZD1236 treatment was provided immediately after injury

Even with a 24‐h delay to treatment after SCI, optimal doses of AZD1236 were equally as effective as immediate delivery, in suppressing SCI‐induced water content (Figure S8A), proinflammatory cytokines (Figure S8B), MMP‐9 and MMP‐12 activity (Figure S8C) and improved electrophysiological (Figure S8D–F), locomotor (Figure S8G) and sensory function (Figure S8H) as well as axon regeneration (Figure S9A,B). At present, we are unsure why delayed treatment is as effective as immediate treatment but believe that there is a time window of therapeutic value of AZD1236 to counteract the negative effects of acute, dysregulated MMP‐9 and MMP‐12 activity.

Since the rat DC injury model better recapitulates human SCI pathophysiology,^1^, ^5^ the same profile of MMP‐9 (Figure S10A) and MMP‐12 (Figure S10B) mRNA expression was also observed. AZD1236 is inactive against rat MMP‐9/‐12, and hence AZD3342, which has similar selectivity to AZD1236, but is active in the rat, demonstrated that SCI‐induced oedema (Figure S10C), MMP‐9 and MMP‐12 activity (Figure S10D), and proinflammatory pain cytokines (Figure S10E) could also be suppressed in the rat DC injury model accompanied by significantly improved electrophysiological (Figure S10F), locomotor (Figure S10G) and sensory function (Figure S10H), similar to that observed with in the mouse SCI model. Finally, since we advocate short‐term inhibition (i.e., the first 3 days after SCI) of MMP‐9 and MMP‐12, we showed that MMP‐9 took 4 days, whilst MMP‐12 took 5 days to return to normal SCI‐induced levels in the injury site, once AZD1236 is withdrawn (Figure S11A,B).

In conclusion, we showed that AZD1236, administered within 24 h after SCI and for only 3 days, promotes unequivocal positive benefits to the key pathophysiological consequences of SCI.^6^, ^7^, ^8^, ^9^, ^10^ AZD1236 suppresses SCI‐induced oedema, BSCB breakdown, neuropathic pain, scarring and infiltration of macrophages into the lesion site while at the same time promoting axon regeneration, leading to improvements in electrophysiological, sensory and locomotor function. This is potentially the first treatment for SCI that is capable of promoting such unprecedented benefits.

## CONFLICT OF INTEREST

Rebecca J. Fairclough is an employee of AstraZeneca UK. Zubair Ahmedis an inventor on a patent related to this work. All other authors declare that they have no competing interests.

## FUNDING INFORMATION

Saudi Arabia Cultural Bureau in London, SHU11, University of Birmingham, Bryant Bequest, ZA1.

## Supporting information

Supporting Information
