# Erratum to “Clinic‐ready inhibitor of MMP−9/−12 restores sensory and functional decline in rodent models of spinal cord injury”

**DOI:** 10.1002/ctm2.70610

**Published:** 2026-02-15

Ahmed Z, Alhajlah S, Thompson AM, Fairclough RJ. Clinic‐ready inhibitor of MMP−9/−12 restores sensory and functional decline in rodent models of spinal cord injury. *Clin Transl Med*. 2022;12(5):e884. https://doi.org/10.1002/ctm2.884.

In Figure 1 M, the panel representing the Caudal DC+Intrathecal AZD1236 group was accidently duplicated for the Rostral DC+Oral AZD1236 group. We have now replaced the Rostral DC+Oral AZD1236 group with a representative image from this group. We stress that this change does not alter any of the conclusions of the originally published paper.

**FIGURE 1:**
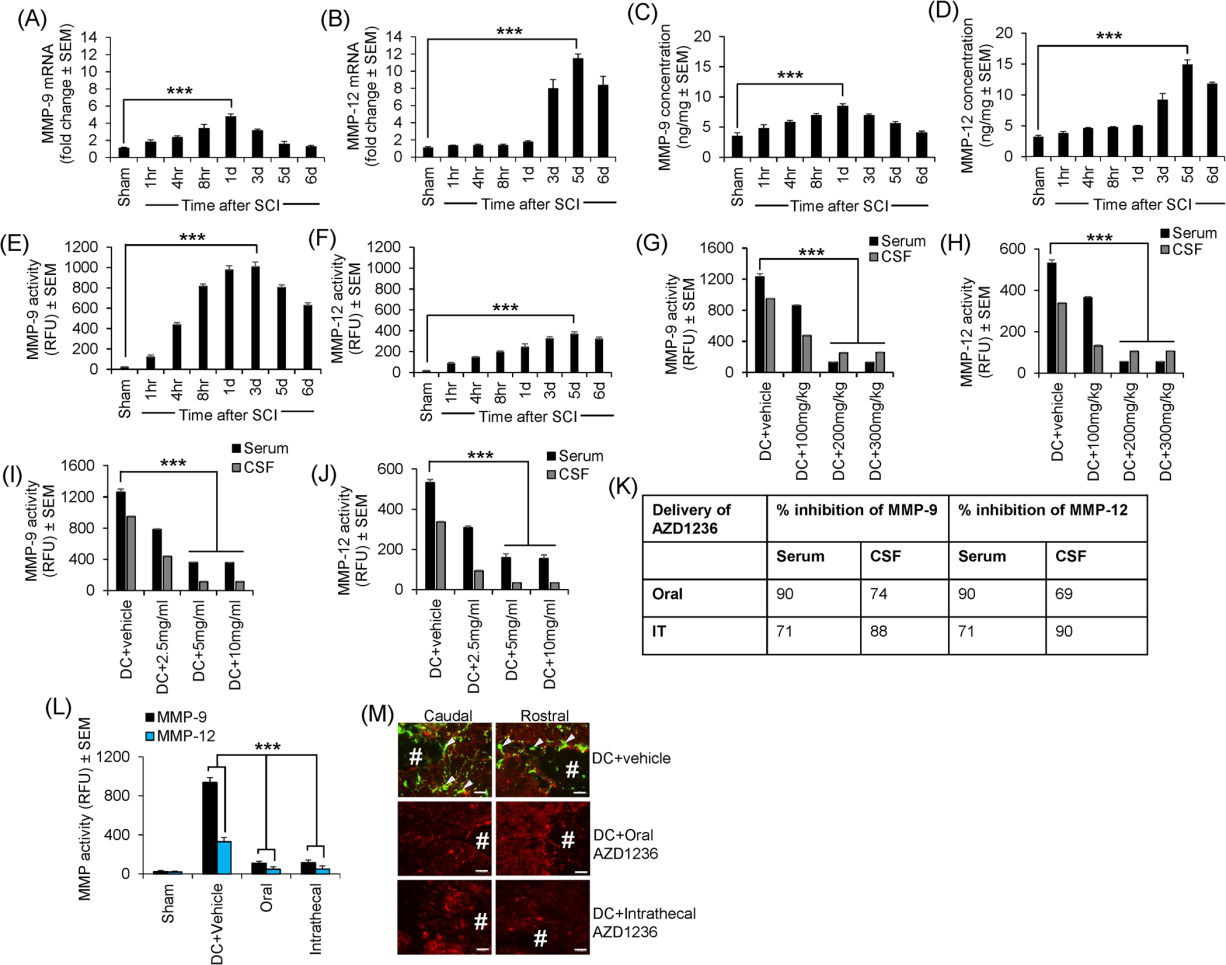
Matrix metalloprotease (MMP)‐9 and MMP‐12 levels and their enzymatic activity increase acutely after dorsal column (DC) injury in mice, and AZD1236 significantly suppresses MMP‐9 and MMP‐12 activity. (A) Levels of MMP‐9 mRNA peak 1 day after injury. (B) Levels of MMP‐12 mRNA peak at 5 days after injury. (C) MMP‐9 protein levels also peak 1 day after injury. (D) MMP‐12 protein levels also peak at 5 days after injury. (E) MMP‐9 activity is high at 1 day and peaks by 3 days after injury. (F) MMP‐12 activity peaks at 5 days after injury. (G) MMP‐9 activity is suppressed by oral delivery of AZD1236 for 3 days after injury in both serum and cerebrospinal fluid (CSF). (H) MMP‐12 activity is also suppressed by oral delivery of AZD1236 in both serum and CSF. (I) MMP‐9 activity is suppressed by intrathecal delivery of AZD1236 for 3 days after injury in both serum and CSF. (J) MMP‐12 activity is also suppressed by intrathecal delivery of AZD1236 in both serum and CSF. (K) Oral delivery of AZD1236 for 3 days after injury suppresses MMP‐9 activity by 90% in both serum and CSF, whilst MMP‐12 is suppressed by 74% and 69% in CSF, respectively. Intrathecal delivery of AZD1236 for 3 days after injury suppresses MMP‐9 activity by 71% in both serum and CSF, whilst MMP‐12 activity is suppressed by 88 and 90% in serum and CSF, respectively. n = 6 mice/group. (L) Optimal doses of AZD1236 also significantly suppress MMP‐9 and MMP‐12 activity in spinal cord homogenates at 3 days after injury. RFU = relative fluorescence units. (M) In situ zymography in saggital sections of the lesion site at 3 days after injury shows that the high levels of gelatinase activity (green; arrowheads) after DC injury is suppressed after oral and intrathecal delivery of optimal doses of AZD1236 in spinal cord sections. Sections are counterstained with GFAP (red) to mark astrocytes in red. # = lesion site. Data are expressed as means ± standard error of the mean (SEM). n = 6 mice/group, two independent experiments, total n = 12 mice/group. p = .0001, one‐way analysis of variance (ANOVA) with Dunnett's post hoc test. Scale bars in (M) = 200 µm. NOTE: AZD1236 treatment was provided immediately after injury.

We apologize for this error.

