# Supplementary material for: Clinic‐ready inhibitor of MMP‐9/‐12 restores sensory and functional decline in rodent models of spinal cord injury
Source: Clin Transl Med. 2022 May 20;12(5):e884. doi: 10.1002/ctm2.884 (PMC9121180; doi:10.1002/ctm2.884)
Supplement: Supplementary file 1 — Supporting Information [file CTM2-12-e884-s001.docx]

**SUPPLEMENTARY INFORMATION FILE**

Clinic-ready inhibitor of MMP-9/-12 restores sensory and functional decline in rodent models of spinal cord injury

Short title: MMP-9 and -12 inhibition in SCI restores function

Zubair Ahmed^1,2,^*, Sharif Alhajlah^1,3^, Adam M. Thompson^1^ and Rebecca J. Fairclough^4^

**MATERIAL AND METHODS**

**Animals**

All animal experiments were licensed by the UK Home Office and ethically approved by the University of Birmingham’s Animal Welfare and Ethical Review Board (approved: 01/09/2015). Experiments were carried out in strict accordance to the guidelines of the UK Animals Scientific Procedures Act, 1986, the Revised European Communities Council Directive (1010/63/EU) and conformed to the guidelines and recommendation of the use of animals by the Federation of the European Laboratory Animal Science Associations and the Animal Research: Reporting of In Vivo Experiments (ARRIVE) guidelines. Wild-type adult male/female C57BL/6 mice (roughly equal proportions), 7-9-week-old and weighing between 20-30g and male/female Sprague-Dawley rats (roughly equal proportions), 6-8-week-old and weighing between 170-220g (purchased from Charles River, Margate, UK) were maintained under a 12-hour light/dark cycle in a pathogen-free facility with controlled temperature and humidity, and were fed ad libitum.

**Study design**

The main objective of this study was to demonstrate that inhibition of MMP-9 and MMP-12 using clinically relevant AZD1236, blocked edema and prevented functional decline in rodent models of SCI. We determined sample sizes based on power calculations in the NC3Rs Experimental Design Assistant (<https://www.nc3rs.org.uk/experimental-design-assistant-eda>) and on our previous similar experiments ^1-5^. In general, all experiments were performed with *n* = 6 animals/group and repeated on 2-3 independent occasions (total *n* = 12-18 animals/group). No animals were excluded for any reason during this study. All animals were randomly allocated to the different experimental groups, ensuring equivalent representation of each animal cage in each experimental group. All treatments were also masked to the investigators and all analysis performed by an experimenter masked to the treatment conditions and only unmasked when analysis was complete.

**Dorsal column (DC) crush and clip compression (CC) models of SCI in mice**

The DC crush injury model of SCI was selected due to being a moderate severity injury that transects the descending corticospinal tract and the ascending sensory gracile and cuneate tracts; the axons of which are derived from pyramidal motor neurons in layer V of the contralateral frontal motor neocortex and ipsilateral dorsal root ganglion neurons (DRGN), respectively. We have previously characterized the injury response after DC lesion in mice ^6^ and others have demonstrated that it is a good model for analysis of regenerative outcomes. However, this model only shows post-injury sensitivity to pain for the first few hours after surgery and cannot be used to assess pain behaviors ^7^. Therefore, we used a more severe, clip compression (CC) model of SCI, described below, to assess pain behaviors after treatment with AZD1236.

***In vivo* experiments**

For oral delivery of AZD1236, *n* = 6 C57BL/6 mice/group (Charles River, Margate, UK) were randomly allocated with the experimenter masked to the treatment conditions, to five groups: (1), Sham (control; partial laminectomy but no DC crush injury); (2), DC crush injury+vehicle (partial laminectomy followed by DC crush injury and injection of vehicle); (3), DC crush injury+100mg/kg AZD1236; (4), DC crush injury+200mg/kg AZD1236; and (5), DC crush injury+300mg/kg AZD1236 (Table S1). For intrathecal (it) delivery of AZD1236, adult male *n* = 6 C57BL/6 mice/group (25-30g) (Charles River, Margate, UK) were also randomly allocated to five groups with the treatment conditions concealed from the experimenters: (1), Sham (control; partial laminectomy but no DC crush injury); (2), DC crush injury+vehicle (partial laminectomy followed by DC crush injury and injection of vehicle); (3), DC crush injury+2.5mg/kg AZD1236; (4), DC crush injury+5mg/kg AZD1236; and (5), DC crush injury+10mg/kg AZD1236. All doses of AZD1236 were selected based on animal and clinical studies performed by AstraZeneca. All doses of other MMP inhibitors were used at concentrations optimized in the literature and as specified in Table S1. Experiments were repeated on at least 2-3 independent occasions (i.e. total *n* = 12-18 animals/group). Animals were orally gavaged with AZD1236 or vehicle, immediately or 8-24 hours after injury, and then twice daily for the duration of each experiment (for 3 days or 4-6 weeks).

For intrathecal (it) delivery, an indwelling atlanto-occipital intrathecal catheter was inserted into the intrathecal space, as described previously ^8^. Briefly, with mice in the sternal recumbency a small incision was made at the nape of the neck followed by detachment of the muscles on either side of the external occipital crest to expose the A-O membrane. The membrane was incised and a 6cm Alzet mouse intrathecal catheter (Alzet, Cupertino, CA, USA) and polyurethane segments were gently guided 2.5cm into the intrathecal space. The catheter was secured using 4-0 silk sutures (Oasis Medical, San Dimas, CA, USA) and the exposed end of the catheter was sealed off with a stainless-steel plug and affixed to the upper back. Animals were injected with immediately or 8-24 h after SCI with vehicle or AZD1236, in a final volume of 10μl followed by a 10μl PBS catheter flush. Injections were repeated twice daily for either 3 days, or 4-6 weeks after injury and drugs and vehicle reagents were delivered over 1 min time period using a Hamilton microlitre syringe (Hamilton Co, USA). All lesions were administered under full general anesthesia using 5% isoflurane, with 1.5l/min O_2_. Pre- and post-injury analgesia was also provided and animals re-housed in their home cages in groups of 4 animals.

DC crush injury was administered bilaterally at the T8 vertebral level as described by us previously ^7,9^. Briefly, calibrated watchmaker’s forceps were separated by 0.5mm and inserted through the dorsal cord meninges to a depth of 0.45mm in mice and separated by 1mm and inserted through the dorsal cord meninges to a depth of 1.0mm in rats, and the DC were crushed for 3 seconds. AZD1236 or vehicle was administered twice daily by oral gavage for the duration of each experiment by experimenters masked to the treatment conditions. All experiments were performed with *n* = 6 mice/group and repeated on 2-3 independent occasions (total *n* = 12-18 mice/group/test).

CC SCI was administered at the T7-T8 vertebral level after exposure of T6-T9 by laminectomy. The aneurysm clip applicator was oriented in a bilateral direction and an aneurysm clip with a closing force of 24g was applied extradurally for 60 seconds, as described previously ^10,11^. The urinary bladders were manually emptied twice daily until bladder function was regained. Adult male C57BL/6 mice (25-30g) (Charles River) were randomly allocated to six groups: (1), Sham (control; laminectomy but no CC); (2), CC+vehicle; (3), CC+200mg/kg AZD1236 (oral); (4), CC+SCI+5mg/kg AZD1236 (it); (5), CC+Pregabalin (oral); (6) CC+Gabapentin (oral). AZD1236 doses preoptimized in the DC crush injury model, as described above were used herein, with the doses masked to the experimenters. Pregabalin and Gabapentin were pre-optimized for oral delivery in the CC model in pilot experiments and used at 30mg/kg and 100mg/kg, respectively. All experiments were performed with *n* = 6 mice/group and repeated on 2-3 independent occasions (total *n* = 12-18 mice/group/test).

Since the rat is considered a more translational model of SCI, the efficacy of MMP-9 and MMP-12 inhibition was also assessed in a rat DC injury model. AZD1236 is not effective against rat MMP-9 and MMP-12 and hence we used AZD3342, another specific inhibitor of MMP-9 and MMP-12 but with activity against rat MMP-9 and MMP-12. The efficacy of oral AZD3342 in the DC injury model in adult Sprague-Dawley rat was assessed. Animals were divided into 6 groups: (1), Sham (control); (2), DC crush injury+Vehicle; (3), DC crush injury +15 mg/kg AZD3342; (4), DC crush injury+75 mg/kg AZD1236; (5), DC crush injury+375mg/kg AZD3342; and (6), DC crush injury+Melatonin. Animals were dosed twice daily with oral AZD3342 or vehicle with the experimenters masked to the treatment conditions. These experiments were performed with *n* = 6 rats/group and repeated on 2-3 independent occasions (total *n* = 12-18 rats/group/test).

**Cholera toxin B labeling of axons**

For retrograde tracing of axons, 1% cholera toxin B (CTB) (#104, List Biologicals Laboratories, Campbell, CA, USA) was injected into the sciatic nerve after exposure at mid-thigh level, using glass microneedles. The skin was re-sutured and the animals allowed to recover for 1 week prior to killing animals using rising concentrations of CO_2_ followed by intracardiac perfusion with 4% paraformaldehyde (TAAB laboratories, Berkshire, UK). CTB labelled axons were detected by immunohistochemistry as described later.

**Quantification of spinal cord water content (oedema)**

The lesion site + 3mm either side for mice and the lesion site + 5mm either side for the rat DC model ^7^ were dissected out and the water content of the spinal cord (as a measure of oedema) was determined at 3 days after DC and CC SCI, as described previously ^5,12^. Spinal cords containing the lesion site were weighed on aluminium foil, dried at 105^o^C for 24 h, and re-weighed. The percent water content was calculated as: water content (%) = [(wet weight-dry weight)/wet weight] x 100%.

**Quantitative RT-PCR (qRT-PCR)**

The lesion site + 5mm either side (DC and CC SCI, *n* = 6 mice/rats/group, 2 independent repeats (total *n* = 12 mice/rats/group)) was dissected and rapidly frozen in liquid N_2_ and stored at -80^o^C until required. Total RNA was extracted from spinal cords at appropriate time-points after injury with or without treatment using TRIzol reagent according to the manufacturer’s instructions (Invitrogen). The levels of mRNA of MMP-9, MMP-12, IL-1β, TNF-α and IL-6 were determined using pre-validated mouse primer sequences from complimentary DNA prepared from extracted mRNA and qRT-PCR was performed using a LightCycler PCR machine (Roche, Burgess Hill, UK) ^9^. Primer sequences for mouse included: MMP-9- cat no. 4331182, Mm0044299_m1; MMP-12- cat no. 4331182, Mm00500554_m1; IL-1β- cat no. 4331182, Mm00434228_m1; TNF-α- cat no. 4331182, Mm00443258_m1; and IL-6- cat no. 4331182, Mm00446190_m1; for rat included: MMP-9 – cat no. 4331182, Rn00579162_m1; MMP-12- cat no. 4331182, Rn00588640_m1; IL-1β- cat no. 4331182, Rn00580432_m1; TNF-α- cat no. 4331182, Rn01525859_g1; and IL-6- cat no. 4881182, Rn01410330_m1 (all from ThermoFisher Scientific, Leicestershire, UK). Fold changes were computed using the ΔΔCt method ^9^.

**Detection of MMP-9 and MMP-12 enzyme activity**

The lesion site + 3mm either side for mouse (DC and CC SCI) and lesion site + 5mm either side for rat was dissected (*n* = 6 mice/rats/group, 2 independent repeats, total *n* = 12 mice/rats/group) and rapidly frozen in liquid N_2_ and stored at -80^o^C until required. The enzymatic activity of MMP-9 and MMP-12 was determined in 96-well plates using the SensoLyte 520 MMP-9 and MMP-12 fluorometric assay kit, according to the manufacturer’s instructions (AnaSpec, Fremont, CA, USA). Fluorescence intensity was measured using a Synergy H1 microplate reader (BioTek UK, Swindon, UK) at Ex/Em = 490/520nm.

***In situ* zymography followed by localization of astrocytes**

Animals were killed by CO_2_ overdose intracardially perfused with warm PBS and unfixed tissues were harvested. The lesion site + 3mm either side for mice and lesion site + 5mm for either side for rat was dissected (*n* = 6 mice/rats/group, 2 independent repeats, total *n* = 12 mice/rats/group) and immediately blocked up in optimal cutting temperature compound (OCT; Miles Inc, Elkhart, IL, USA) and stored at -80^o^C until required for cryostat sectioning. *In situ* zymography followed by immunolocalization of cell markers was performed as described by us previously ^13^. Briefly, 15µm-thick unfixed frozen longitudinal sections of the spinal cord were cut on a cryostat and incubated at 25^o^C for 24 h in 50 mM Tris, pH 7.4, 150 mM NaCl, 5 mM CaCl2, 0.2 mM sodium azide and 40 ug/ml fluorescein conjugated DQ^TM^ gelatin (Invitrogen). Upon proteolysis, the highly fluoresceinated gelatin residues became separated and fluorescence resulted. Control sections were incubated either without DQ^TM^ gelatin or incubated with 50 µM 1,10-phenanthroline (Sigma, Poole, UK) to block MMP activation. Sections were then fixed in 4% paraformaldehyde (TAAB Laboratories, Berkshire, UK), washed in PBS and incubated with anti-glial fibrillary acidic protein (GFAP) antibodies (SAB5700611, 1:400 dilution, GFAP; Sigma, Poole, UK) for 1 hour at room temperature in a humidified chamber. Sections were then washed in PBS and incubated with appropriate secondary antibodies conjugated with Texas Red (Invitrogen, Paisley, UK). After final washes in PBS, sections were mounted in Vectashield with DAPI (Vector Laboratories, Loughborough, UK) and viewed under a Zeiss Axioplan 2 fluorescence microscope, equipped with an AxioCam HRc and running Axiovision software (all from Zeiss, Hertfordshire, UK).

**Immunohistochemistry**

After killing animals by exposure to rising concentrations of CO_2_, mice were intracardially perfused with 4% formaldehyde (TAAB Laboratories) in 0.1M phosphate buffered saline (PBS). The lesion site + 5mm either side were dissected out, post-fixed in 4% formaldehyde and subjected to a graded series of sucrose solutions for cryoprotection ^7^. Spinal cords were blocked up in OCT mounting compound (TAAB laboratories), sectioned longitudinally at 15μm-thick using a cryostat (Brights Instruments, Huntingdon, UK) before being collected on charged glass slides (ThermoFisher Scientific) and kept at -20^o^C until required. Slides were numbered consecutively and sections from the middle of the lesion site were chosen for all immunohistochemical analyses, as described by us previously ^6,7^.

The integrity of the BSCB was assessed using albumin immunoreactivity in the spinal cord at 3 days after injury, as a surrogate marker of BSCB disruption ^14-17^. Sections were thawed at room temperature and washed in PBS before blocking endogenous peroxide in H_2_O_2_. Sections were then permeabilization in 0.1% Triton X-100 in PBS for 10 min at room temperature, blocked in 4% serum in PBS and incubated overnight with rabbit anti-albumin primary antibodies (ab271979; 1:1000 dilution; Abcam, Cambridge, UK). Sections were then washed in PBS and incubated for 1 h at room temperature with HRP-labelled anti-rabbit secondary antibody, followed by washes in PBS and incubation with avidin-biotin conjugate for 30 min. Color was developed using the 3,3’-Diaminobenzidine (DAB) substrate kit, dehydrated through a graded series of alcohols, cleared in Histoclear and coverslips mounted in Vectamount (all from Vector Labs).

Fluorescent immunohistochemistry was performed as described by us previously ^7^. Briefly, longitudinal spinal cord sections were washed in PBS, followed by incubation in PBS containing 1% (v/v) Triton X-100 (Sigma) to permeabilize cells. Sections were then blocked for 30 min at room temperature (RT) using PBS containing 0.05% (w/v) bovine serum albumin (Sigma, Poole, UK) and 0.05% Tween-20 (Sigma) and incubated overnight at 4°C in a humidified chamber with appropriate primary antibodies. Laminin was detected at 4 weeks after DC injury and treatment using a rabbit polyclonal anti-laminin primary antibody (ab11575; 1:400 dilution, Abcam). Macrophages were detected with a rabbit polyclonal anti-CD68 antibody (ab1525212; 1:500 dilution, Abcam); microglia were detected using a rabbit polyclonal antibody to CD11b (ab128797, 1:500 dilution, Abcam); GFAP was detected using a polyclonal anti-GFAP antibody (SAB5700611, 1:400 dilution, Sigma) were all detected at 10 days after DC injury and treatment. Semaphorin-3A (Sema-3A) and chondroitin sulphate proteoglycan (CSPG) was detected using monoclonal anti-CS-56 antibody (C8035; 1:200 dilution, Sigma) at 7 days after injury. We chose this timepoint since a number of studies have indicated that CSPGs are observed around the lesion site at 7 days after injury ^18-20^. CTB labelled axons were detected using a goat polyclonal anti-CTB antibody (#703; 1:1000 dilution, List Biological Labs) at 6 weeks after DC injury.

Sections were then washed in PBS before incubation for 1 h at room temperature with appropriate Alexa488 and Alex595-conjugated secondary antibodies (all used at 1:400 dilution; Invitrogen). Finally, sections were washed in PBS and coverslips mounted using Vectashield mounting medium (containing DAPI) (Vector Laboratories). Negative controls were included in each run where primary antibodies were omitted and these slides were used to set the background threshold levels prior to image capture. All image capture and analysis were performed by an investigator masked to the treatment conditions. Sections (fluorescent and DAB stained) were viewed using and Axioplan 2 fluorescent microscope equipped with an AxioCam HRc and Axiovision software (Zeiss).

**Quantification of immunofluorescence**

All analyses were performed by investigators masked to the experimental groups. Relative fluorescent staining intensity was calculated by image analysis as described by us previously ^7^. Briefly, photomicrographs taken at 5x magnification using the same standardized exposure settings throughout for each antibody were thresholded and the mean integrated intensity of pixels/unit area for each antibody was recorded Using ImageJ (NIH, USA) from *n* = 12 mice/antibody.

Regeneration of DC axons were quantified from sagittal spinal cord sections form the whole series of tissue for each mouse (total *n* = 12 mice/group). CTB intensity was quantified using ImageJ software at set distances rostral to the injury center and expressed as a percentage of the CTB intensity caudal to the injury site to control for variations in tracing efficiency.

**Primary microglia cultures and treatments**

Primary mouse microglia from 6-8-week-old C57BL/6 mouse brains were prepared according to a previously described protocol ^21^. Briefly, the meninges of brains were dissected out, finely minced enzymatically digested using 20 units/ml Papain (all from Sigma). After incubation for 90 min at 37^o^C, the suspension was centrifuged at 200 g for 7 min and the pellet resuspended in 0.5mg/ml DNase I (Roche, Manheim, Germany) and triturated with fire-polished Pasteur pipettes of decreasing diameters. The homogenate was then filtered through a 70µm cell strainer (Beckton Dickinson, Watford, UK), and centrifuged through a Percoll (GE Healthcare, Amersham, UK) gradient. Finally, the cell suspension was resuspended in DMEM/F12 medium supplemented with 10% FBS (all from Invitrogen) and 5ng/ml of granulocyte colony and macrophage stimulating factor (GM-CSF) (#415-ML, R&D Systems. Watford, UK), plated out in T75 cell culture flasks (Beckton Dickinson) precoated with poly-L-lysine, and maintained at 37^o^C and 5% CO_2_ for approximately 2 weeks. When the cells become confluent, microglia detach and migrate to the medium-air interface, floating and proliferating. The supernatant of the flask was then collected without prior shaking to remove the microglia growing on a mixed glial culture base and centrifuged at 200 g for 7 min. The purity of microglia was determined from each flask by immunocytochemistry for CD11b and confirmed as 95% pure. Before each experiment, microglia were cultured in DMEM/F12 without GM-CSF for at least 3 d.

Microglia were used for all experiments with an *in vitro* age between 15-20 days *in vitro* (DIV) and were seeded on glass coverslips in 24-well plates at a density of 3 x 10^4^ cells/well. Microglia were exposed to serum-free media for 24 h before exposing the cells to different concentrations of lipopolysaccharide (LPS) in preliminary experiments to determine optimal concentrations required to maximally activate our primary microglial cultures. This was determined as 10ng/ml. For the microglial activation assay, cells were exposed to serum-free medium for 24 h before being exposed to LPS, with or without different concentrations of AZD1236 (10-500ng/ml) for a further 24 h. Cell culture supernatant was then collected, centrifuged to remove cell debris and subjected to ELISA to determine the concentrations of TNF-α, IL-1β and IL-6. Experiments were performed in triplicate wells and repeated on 3 independent occasions (*n* = 9 wells/treatment).

**Enzyme-linked immunosorbent assay (ELISA)**

The levels of TNF-α (#MTA00B), IL-1β (#MLB00C) and IL-6 (#M6000B) were determined using commercially available kits from R&D Systems, following the manufacturer’s instructions.

To detect levels of CSF biomarkers with and without AZD1236 treatment, 20µl of CSF was harvested from each animal in terminal experiments at 3 days after injury and treatment from the right lateral ventricle as described previously ^22^. CSF samples were centrifuged to remove potential contaminants and the levels of S100β, neuron specific enolase (NSE; MBS028924, My BioSource, San Diego, USA), glial fibrillary acidic protein (GFAP; abx155574, Abbexa Ltd, Cambridge, UK), phosphorylated neurofilament-H (pNF-H; DEIA7367, Creative Diagnostics, London, UK) and neurofilament light chain (NF-L; MBS9399608, My BioSource) were detected using commercially available kits, according to the manufacturer’s instructions.

**Cell culture**

J774A.1 cells (#TIB-67; ATCC, Middlesex, UK) were cultured in DMEM supplemented with 10% FBS, 100 U/ml penicillin, 100 μg/ml streptomycin, and 0.25 μg amphotericin B. Cells were seeded in T75 tissue-culture flasks and maintained at 37°C and 5% CO_2_. RAW 264.7 cells (#TIB-71; ATCC) were maintained in DMEM containing 10% FBS.

**Peritoneal macrophage preparation**

Resident peritoneal cells were harvested from adult C57BL/6 mice (6-8 weeks old) by injection of 10 ml of PBS using a 21G needle into the peritoneal cavity and harvesting the cell suspension, as described previously ^23^. Peritoneal cells were then centrifuged at 100 x g for 10 min and the cell pellet resuspended in DMEM/F12 medium. Cell were incubated at 37^o^C for 2 h and non-adherent cells were removed by gentle washing. Cells were expanded in DMEM/F12 medium containing 10% FBS. For migration assays, cells were grown in RPMI containing 0.02% BSA for 24 h to obtain quiescent cells prior to using them in Transwell migration assays as described below.

**Transwell migration assays**

Migration assays were performed with J774A.1, RAW 264.7 and primary mouse peritoneal macrophages in 6.5 mm Transwell plates (ThermoFisher) with 8 μm pore inserts, as described previously ^24^. Briefly, inserts were coated with rat tail type I collagen and 1 x 10^5^ cells (J774A.1, RAW 264.7 or primary mouse macrophages) were resuspended in chemotaxis buffer (RMPI 1640 plus 0.02% BSA, (termed RPMI from herein)) and added to the upper chamber and incubated with migration medium, with or without known chemotactic factors MCP-1 (100 ng/ml) and PMA (100 nM), or AZD1236 (1, 10, 100, 1000ng/ml) added to the lower chamber. Cells were allowed to migrate through the insert membrane for 3 h at 37°C, before washing inserts with PBS. Non-migrating cells remaining on the upper surface were removed with a cotton swab whilst the migrated cells on the insert were fixed, stained with Diff-Quick (#26096, Electron Microscopy Science, Hatfield, UK), and mounted on glass slides. Migration was measured visually by counting using a light microscope at 40× magnification. The mean number of cells in 10 random fields were calculated for each treatment by an experimenter masked to the treatment conditions. A migration index was calculated by dividing the number of cells that migrated in response to the chemokine by the number of cells that migrated randomly (RPMI medium) with a reference index >1, indicating chemotaxis.

**Electrophysiology**

Compound action potentials (CAP) were recorded at 6w weeks after DC injury and treatment, as described previously ^1,25,26^. Briefly, the experimenter was masked to the treatment status of the animals and the CAP amplitude was calculated between the negative deflection after the stimulus artifact and the next peak of the wave. CAP area was also calculated by rectifying the CAP component (full-wave rectification) and measuring its area. To confirm our recordings and that a CAP could not be recorded, the dorsal half of the spinal cord was transected after each experiment between the stimulating and recording electrodes.

**Functional tests**

Functional testing after DC lesion and treatment was carried out as described previously ^1,4,5,27^. Briefly, animals (*n* = 18/group) were first trained to master traversing a horizontal ladder for 1 week before functional testing. Baseline parameters were established by performing tests at 2-3 days before injury. Animals were then tested at 2 days, 1 week, 2 weeks, 3 weeks, 4 weeks, 5 weeks and 6 weeks after DC injury+treatment. Experiments were performed by an observer masked to the treatment conditions in the same order and time of day with each test performed for 3 individual trials.

*Horizontal ladder test:* This tests the animals locomotor function and is performed on a 0.9-meter-long horizontal ladder with a diameter of 15.5cm and randomly adjusted rungs with variable gaps of 3.5-5.0cm. Animals were assessed traversing the ladder and the left and right rear paw slips were recorded along with the total number of steps and the mean error rate as: the number of slips/total number of steps.

*Tape sensing and removal test (sensory function):* The tape removal test determines touch perception from the left hind paw. After holding animals with both hind-paws extended, the time it took for the animal to detect and remove a piece of tape of 15x15mm (Kip Hochkrepp, Bocholt, Germany) affixed to the palm of the left hind-paw was recorded and used to calculate the mean sensing time.

**Assessment of neuropathic pain**

*Mechanical allodynia* was measured in mice using a series of von Frey filaments (0.25g-15g) applied to the plantar surface of the hind-paw by an experimenter masked to the treatment conditions. Withdrawal of the paw was recorded as a positive response ^28^ and the hair with the lowest force required to elicit a paw withdrawal response was recorded. Both hind paws were tested with a 5 min rest between testing of the opposite paw.

*Thermal hyperalgesia* was tested using established methods of plantar heat sensitivity tests by an experimenter masked to the treatment conditions, as described by us previously ^7^. Briefly, animals were acclimatized in clear Perspex compartments before surgery post injury and treatment data were collected at the same time of day to ensure consistency. Animals were also acclimatized to the clear Perspex compartments for 5 min before testing commenced. An infra-red heat source (Harvard Apparatus, Kent, UK) was applied to the plantar surface of the hindlimbs once the animals were stationary. The reaction time of paw withdrawal was recorded for five separate tests for each hind limb with a 30 second interval before re-testing on the same paw. The middle three scores for the hind limbs were averaged to produce a single score for each animal.

*Cold allodynia* was measured by an experimenter masked to the treatment conditions as the number of foot withdrawal responses after application of an acetone drop to the plantar surface of the paw ^28^. Testing was repeated five times with an interval of 5 min between each test and the response frequency to acetone was expressed as a percent response frequency ([number of paw withdrawals/number of trials] x 100).

**Statistical analysis**

Statistical significance was calculated from sample means by one-way analysis of variance (ANOVA) with post-hoc Dunnett’s method using SPSS Statistics 19 (IBM, New York, USA). For the horizontal ladder crossing and tape removal tests, data was analysed as described previously ^1,4,5,27^ using R package ([www.r-project.org](http://www.r-project.org)). Briefly, for the ladder crossing test, whole time-course of lesioned and sham-treated animals were compared using binomial generalized linear mixed models (GLMM). Binomial GLMMs were fitted in R using package *lme4* with the *glmer* function. P values were then calculated using parametric bootstrap. For the tape removal test, linear mixed models (LMM) were calculated by model comparison in R using the package *pbkrtest*, with the Kenward-Roger method ^1,4,5,27^. For pain behavior tests, data were compared among groups using two-way ANOVA with repeated measures followed by Bonferroni *post hoc* tests. All results are presented as mean ± standard error of the mean (SEM) and error bars in the figures represent SEM.

**SUPPLEMENTARY FIGURES**

**
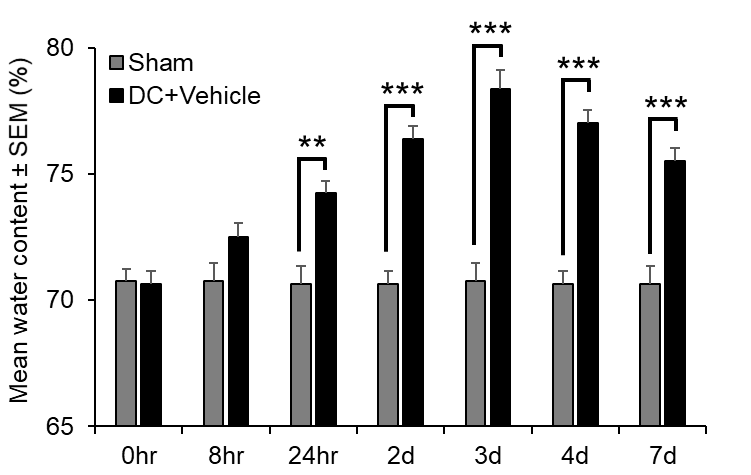
**

**FIGURE S1.** **Mouse spinal cord water content.** The mean water content (oedema) in the spinal cord rises and peaks at 3 days after DC injury and thereafter declines, compared to sham-treated control levels. *n* = 6 mice/group, 2 independent experiments, total *n* = 12 mice/group. ** = *P* = 0.001; *** = *P*=0.0001, one-way ANOVA with Dunnett’s *post hoc* test. NOTE: AZD1236 treatment was provided immediately after injury.


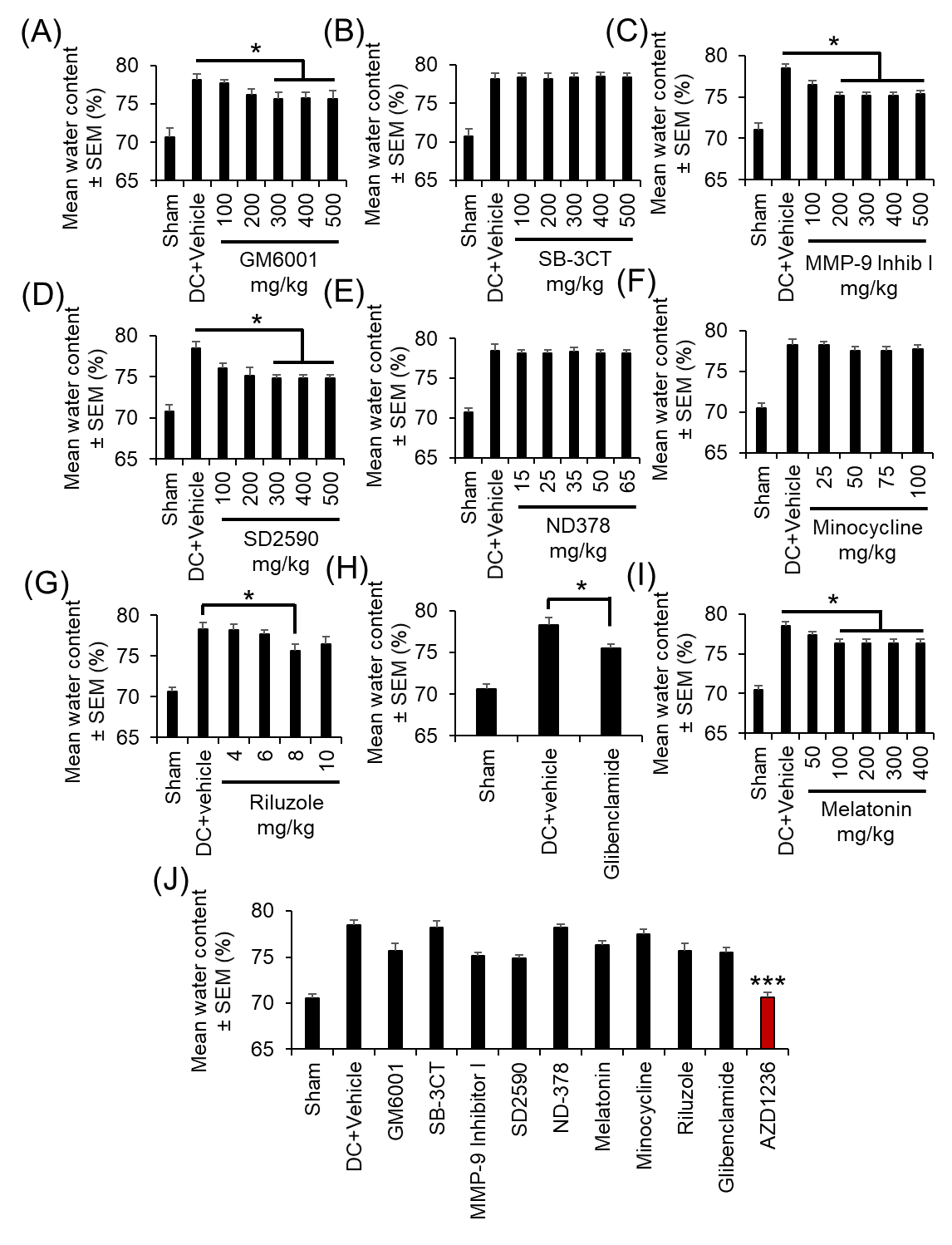


**FIGURE S2.** **Comparison of MMP inhibitors and their ability to suppress water content in the spinal cord at 3 days after DC injury in mice.** (**A**) Mean water content of the spinal cord after treatment with GM6001. * = *P* = 0.05, one-way ANOVA with Dunnett’s *post hoc* test. (**B**) Mean water content of the spinal cord after treatment with SB-3CT. (**C**) Mean water content of the spinal cord after treatment with MMP-9 Inhibitor I. * = *P* = 0.05, one-way ANOVA with Dunnett’s *post hoc* test. (**D**) Mean water content of the spinal cord after treatment with SD2590. * = *P* = 0.05, one-way ANOVA with Dunnett’s *post hoc* test. (**E**) Mean water content of the spinal cord after treatment with ND378. (**F**) Mean water content of the spinal cord after treatment with Minocycline. (**G**) Mean water content of the spinal cord after treatment with Riluzole. * = *P* = 0.05, one-way ANOVA with Dunnett’s *post hoc* test. (**H**) Mean water content of the spinal cord after treatment with Glibenclamide. * = *P* = 0.05, one-way ANOVA with Dunnett’s *post hoc* test. (**I**) Mean water content of the spinal cord after treatment with Melatonin. * = *P* = 0.05, one-way ANOVA with Dunnett’s *post hoc* test. (**J**) Compared to all MMP inhibitors used in this study, AZD1236 was far superior, almost completely ablating DC injury-induced rises and suppressing these levels back to sham-treated control levels. *** = *P*=0.00012, one-way ANOVA with Dunnett’s *post hoc* test. Data are expressed as means ± SEM. *n*= 6 mice/group, 2 independent experiments, total *n* = 12 mice/group. * = *P* = 0.05, one-way ANOVA with Dunnett’s *post hoc* test. NOTE: AZD1236 and other MMP inhibitors were given immediately after injury.


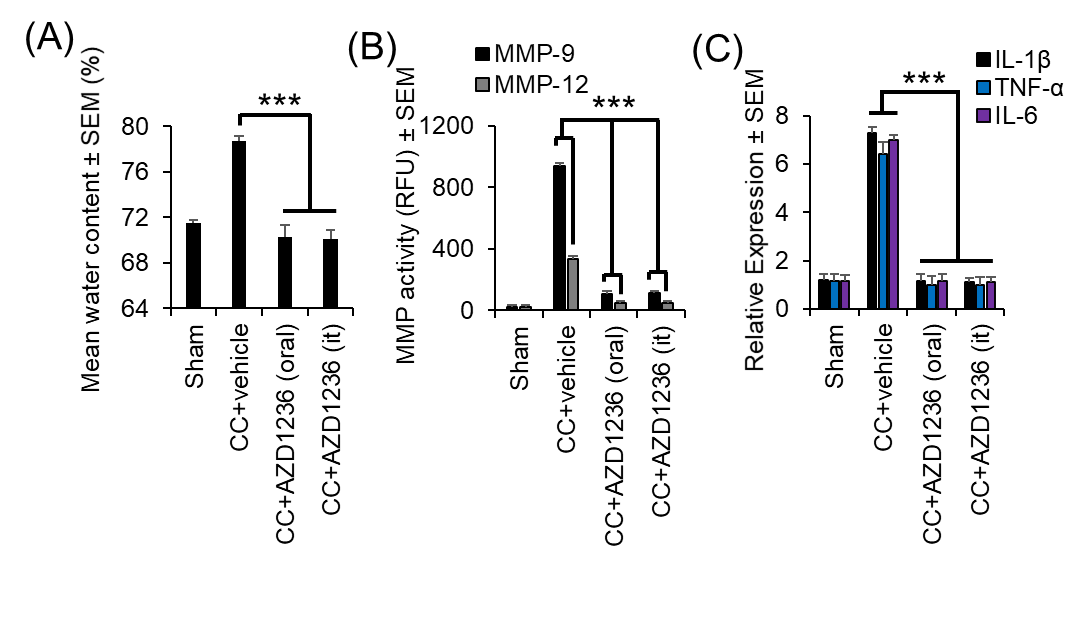


**FIGURE S3. Inhibition of MMP-9 and MMP-12 also suppresses SCI-induced edema, proinflammatory cytokines and MMP activity in a clip compression (CC) model in mice.** (**A**) Water content is reduced by inhibition of MMP-9 and MMP-12 by both oral and intrathecal AZD1236. (**B**) The levels of mRNA for proinflammatory cytokines is also reduced in a CC model. (**C**) AZD1236 significantly attenuates MMP-9 and MMP-12 activity after oral and intrathecal delivery in the CC model. Data are expressed as means ± SEM. *n* = 6 mice /group, 2 independent repeats, total *n* = 12 mice/group. *** = *P*=0.0001, one-way ANOVA with Dunnett’s post hoc test. NOTE: AZD1236 treatment was provided immediately after injury.


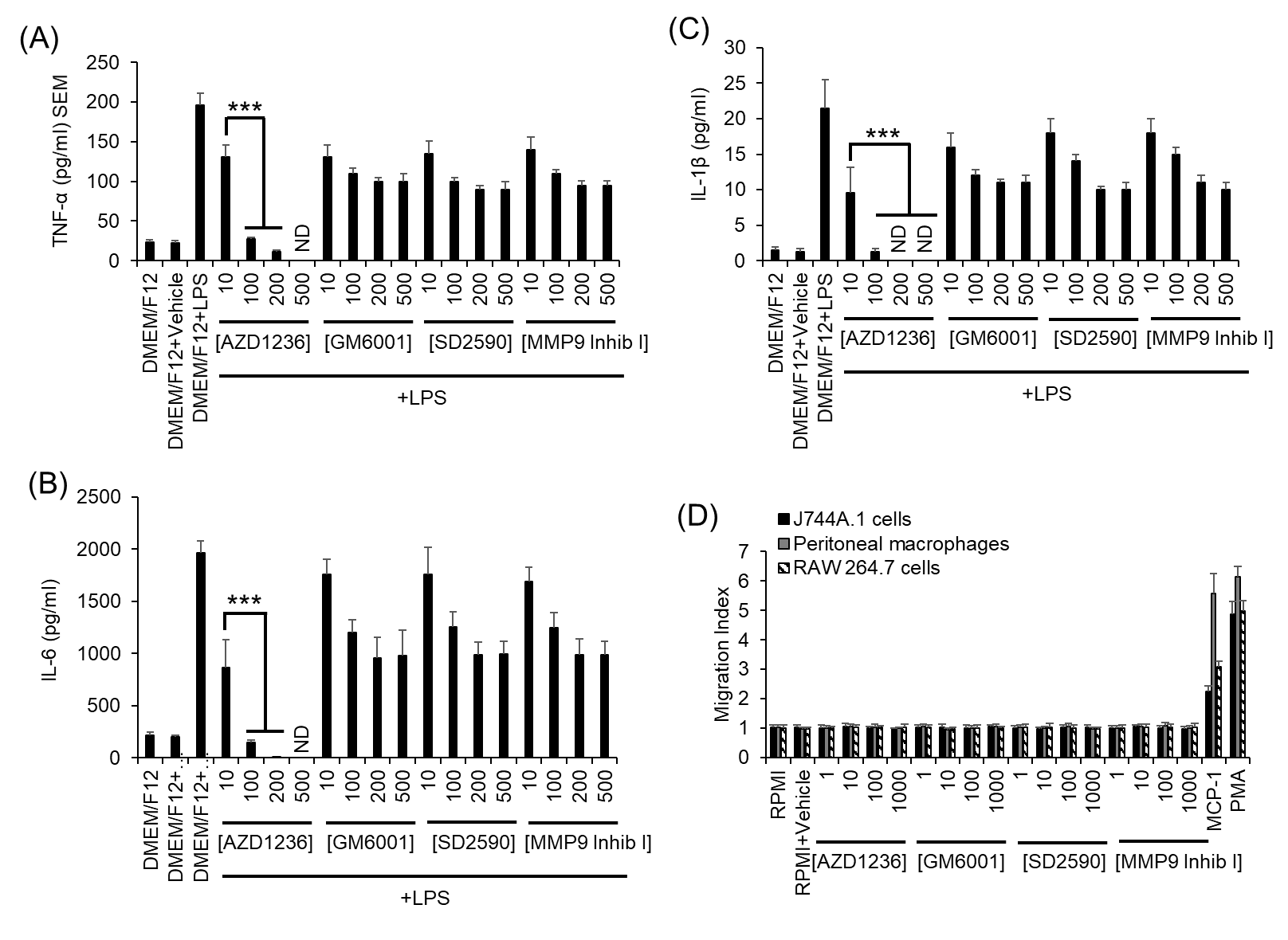


**FIGURE S4.** **AZD1236 attentuates proinflammatory cytokine release from LPS-stimulated microglia but does not affect macrophage migration in vitro.** (A) TNF-α, (B) IL-1β and (C) IL-6 production by LPS-stimulated primary microglia is inhibited by AZD1236. In comparison, other MMP inhibitors such as GM6001, SD2590 and MMP Inhibitor I only marginally attentuate TNF-α, IL-1β and IL-6 levels. (D) Macrophage migration is not affected by AZD1236, GM6001, SD2590 or MMP9 Inhibitor I but postive controls, MCP-1 and PMA, signficantly increase migration index in all macrophage populations. *n* = 3 wells/treatment, 3 independent repeats (total n = 9 wells/treatment). ** = *P*<0.001; *** = *P* <0.0001, one-way ANOVA with Dunnett’s post hoc test. NOTE: AZD1236 treatment was provided immediately after injury.

**
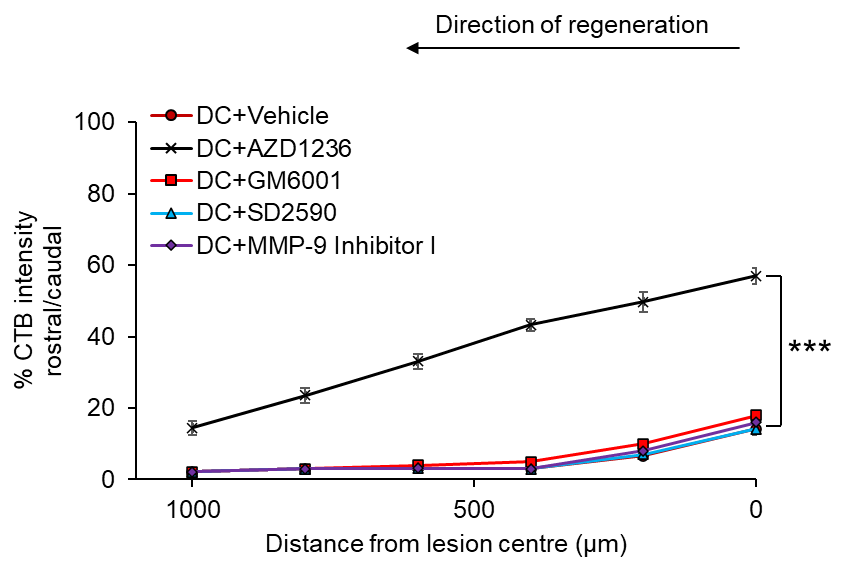
**

**FIGURE S5. AZD1236 promotes significantly more DC axon regeneration after DC injury in mice than other tool MMP inhibitors such as GM6001, SD2590 and MMP-9 Inhibitor I**. *n* = 12 nerves/treatment. *** = *P* <0.0001, one-way ANOVA with Dunnett’s post hoc test. NOTE: AZD1236 treatment was provided immediately after injury.


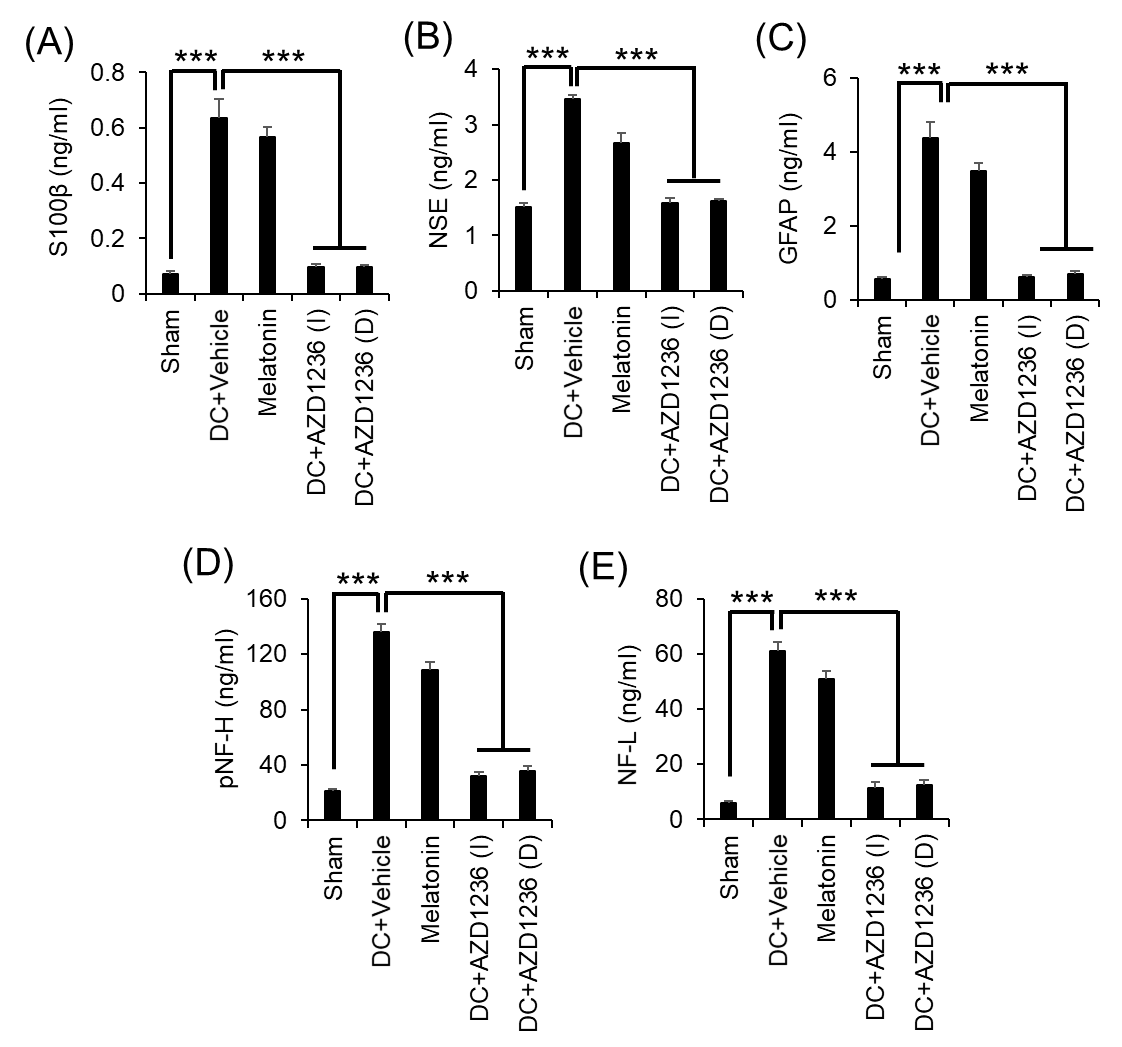


**FIGURE S6. Common CSF biomarkers of SCI are also attenuated by AZD1236 in mice.** (**A**) S100β, (**B**) NSE, (**C**) GFAP, (**D**) pNF-H and (**E**) NF-L are all marginally reduced by Melatonin treatment but significantly attenuated by AZD1236. *n* = 12 mice/group. *** = *P*=0.0001, one-way ANOVA with Dunnett’s post hoc test. NOTE: AZD1236 (200mg/kg oral) treatment was provided immediately after injury.

**
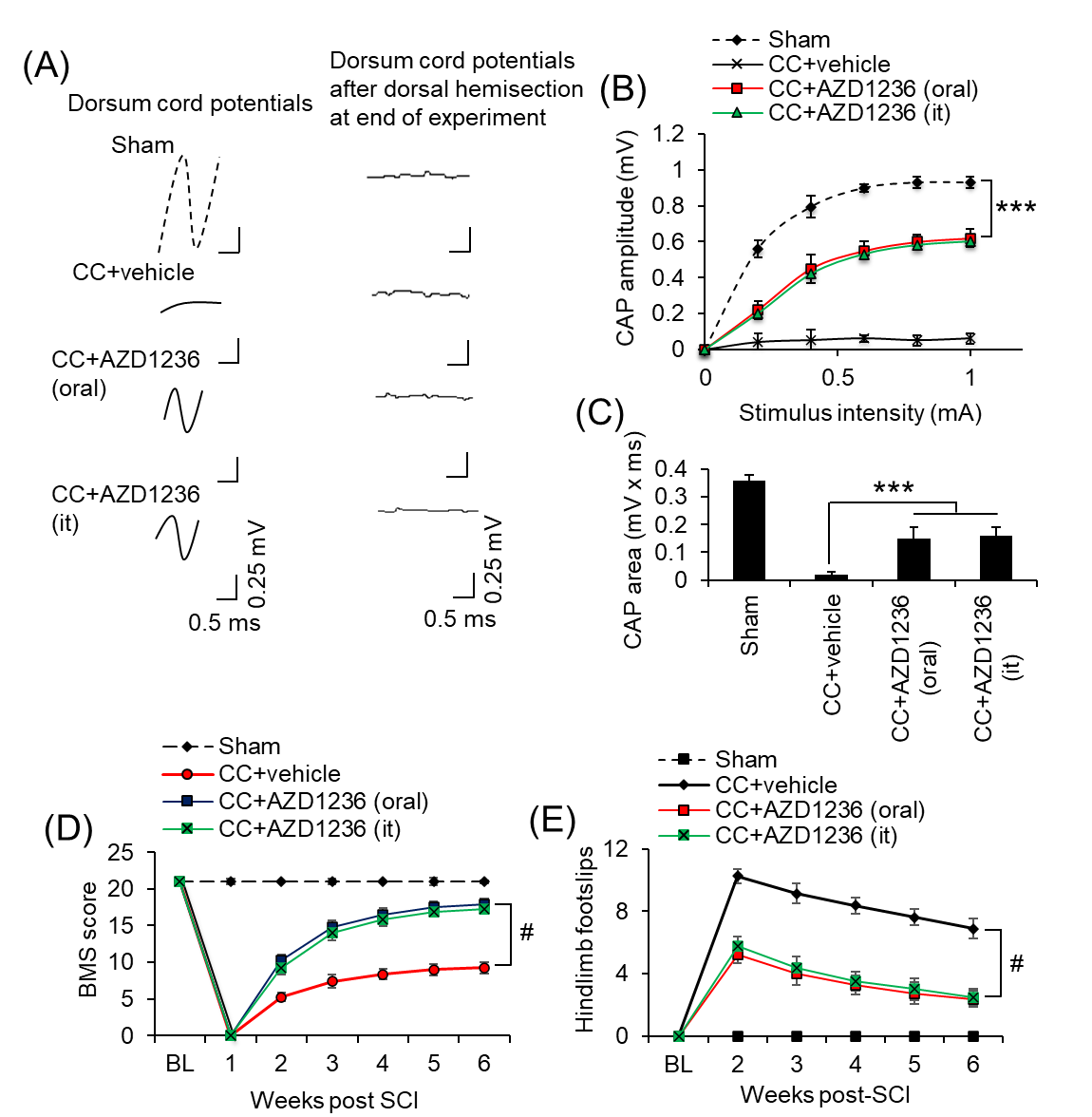
**

**FIGURE S7. AZD1236 treatment improves electrophysiological and locomotor recovery after severe CC SCI in mice.** (**A**) AZD1236 treatment restored a significant CAP trace after injury and delivery by either oral or intrathecal (it) routes. AZD1236 also significantly improved (**B**) CAP amplitudes and (**C**) CAP areas. In addition, locomotor performance as assessed by the (**D**) Basso Mouse Scale and (**E**) ladder crossing was also significantly improved by oral and intrathecal delivery of AZD1236. *n* = 12 mice/group. ** = P=0.001; *** = *P*=0.0001, one-way ANOVA with Dunnett’s post hoc test. # = *P*=0.00012, linear mixed models. NOTE: AZD1236 treatment was provided immediately after injury.


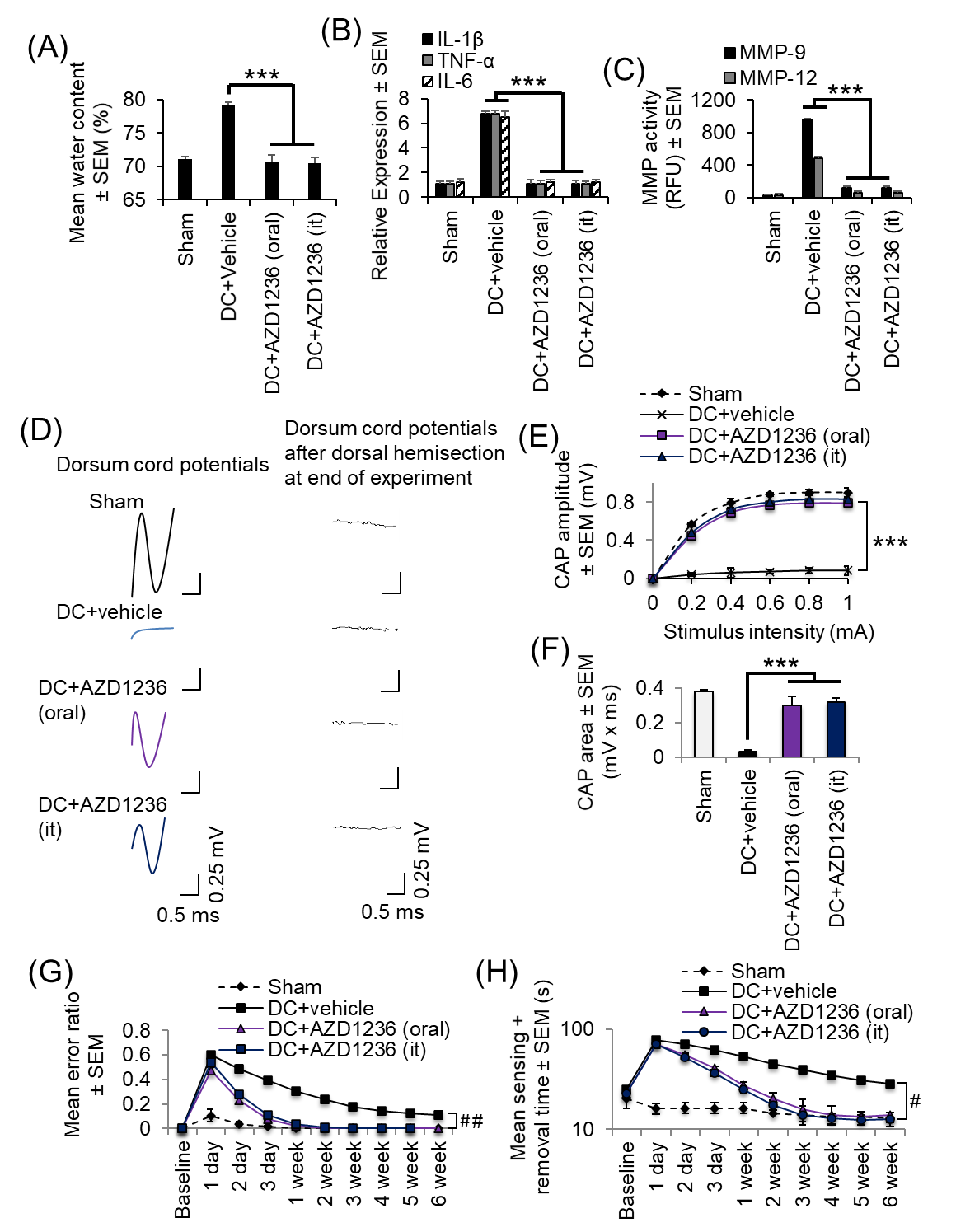


**FIGURE S8.** **Twenty-four hour delayed treatment with AZD1236 is as beneficial as immediate treatment in mice.** (**A**) Data to show that AZD1236 significantly suppressed SCI induced spinal cord water content. *** = *P*=0.0001, one-way ANOVA with Dunnett’s post hoc test. (**B**) Expression of proinflammatory pain markers are also suppressed. *** = *P*=0.0001, one-way ANOVA with Dunnett’s post hoc test. (**C**) MMP-9 and MMP-12 activity is also suppressed by AZD1236. *** = *P*=0.0001, one-way ANOVA with Dunnett’s post hoc test. (**D**) Representative Spike 2 processed CAP waves after delayed treatment with AZD1236. (**E**) Significant improvements in CAP amplitudes after treatment with AZD1236. *** = *P*=0.0001, one-way ANOVA with Dunnett’s post hoc test. (**F**) CAP areas were also improved by AZD1236 treatment. *** = *P*=0.0001, one-way ANOVA with Dunnett’s post hoc test. (**G**) Ladder crossing and (**H**) sensory function test both showed signficant improvements. Data are expressed as means ± SEM. ## = *P*<0.0012, generalized linear mixed model. # = P<0.0011, linear mixed models. *n* = 6 mice/group, 2 independent experiments, total *n* = 12 mice/group. NOTE: AZD1236 treatment was provided at 24 hours after injury.


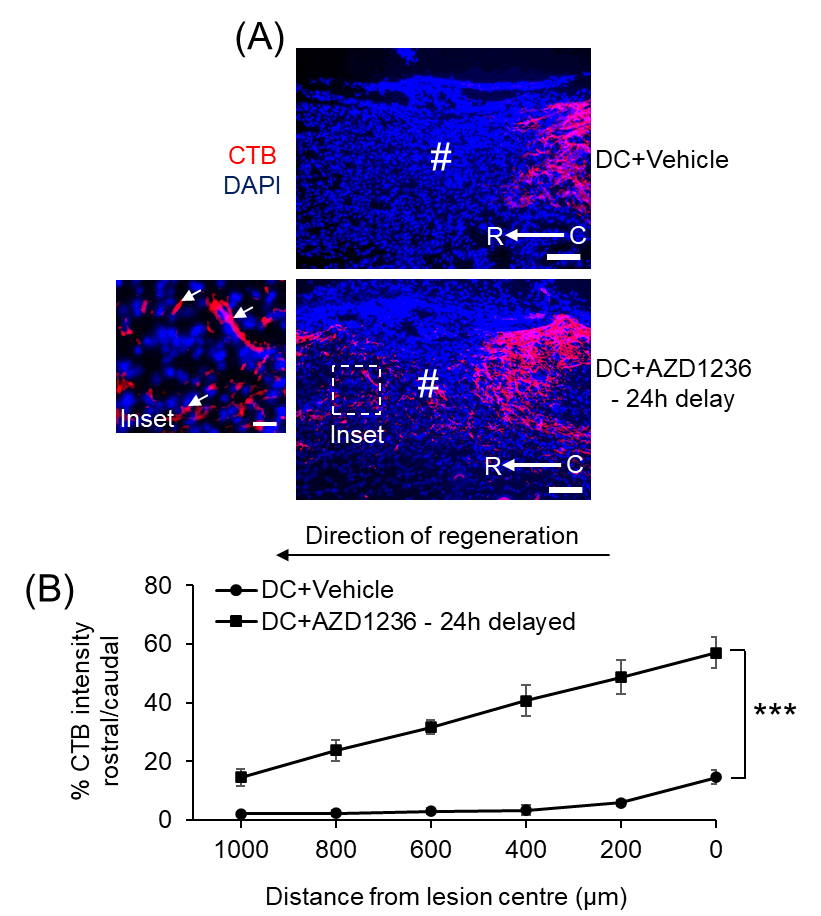


**FIGURE S9.** **Twenty-four hour delayed treatment with AZD1236 promotes similiar proportions of axon regeneration as immediate treatment in mice.** (**A**) CTB^+^ axons stopped at the lesion site (#) in DC+Vehicle-treated mice whereas, signficant proportions of CTB^+^ lablled axons were observed regenerating through the lesion site and entering the rostral cord in animals treated with AZD1236. C = caudal, R = Rostral. Scale bars = 200µm. (**B**) Quantification of the proportion of CTB^+^ regenerating axons shows similar proportions of axons as immediate treatment. *** = *P*=0.0001, one-way ANOVA with Dunnett’s post hoc test. *n* = 6 mice/group, 2 independent repeats (total *n* = 12 mice/group). NOTE: AZD1236 treatment was provided 24 hours after injury.

**
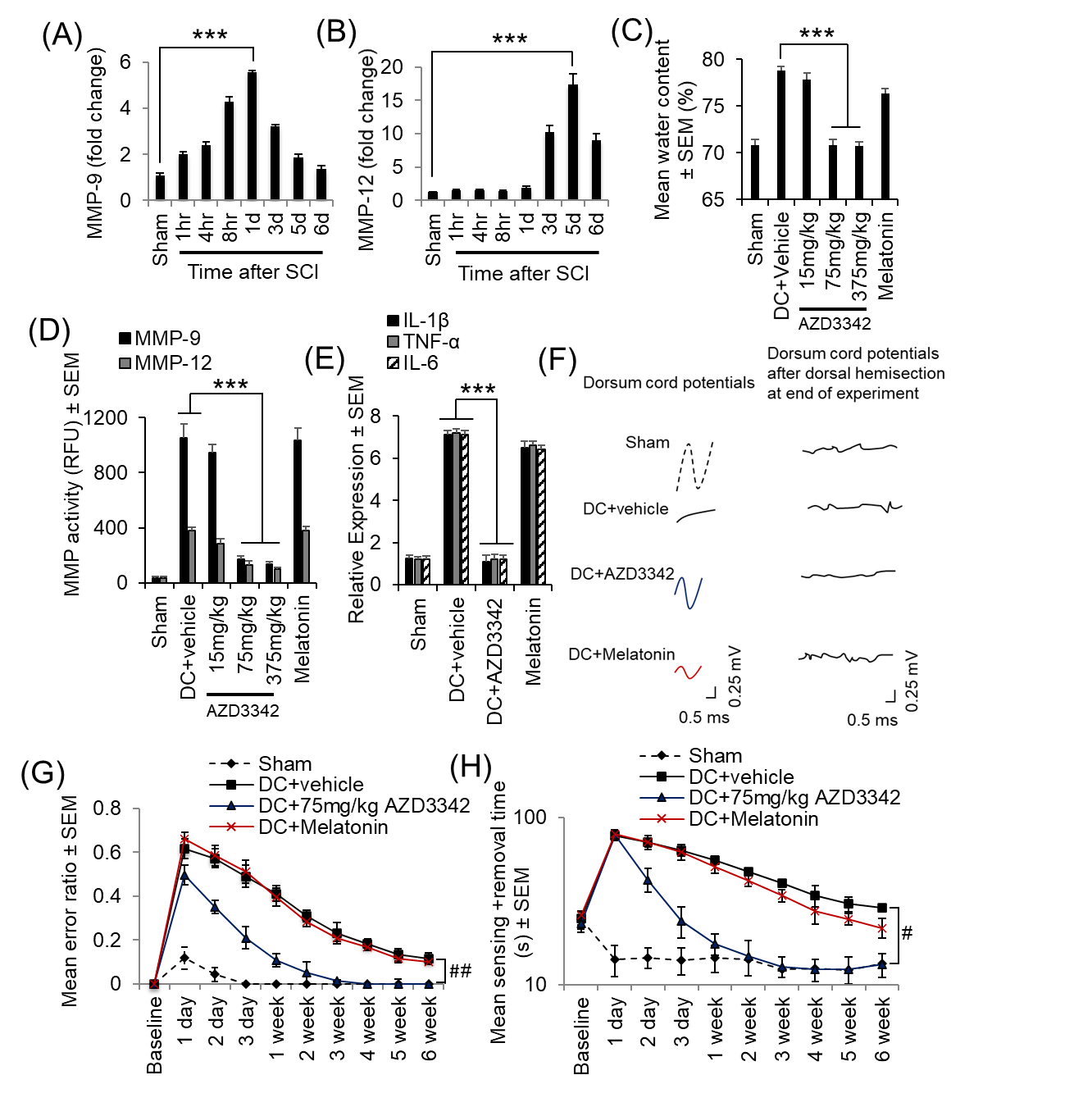
**

**FIGURE S10.** **Inhibition of MMP-9 and MMP-12 using AZD3342 is also effective in rat models of DC injury.** (A) MMP-9 mRNA levels in the rat also peak at 1 day after DC injury. (B) MMP-12 mRNA levels in the rat also peak at 5 days after DC injury. (C) Spinal cord water content (edema) is ablated in rats at 3 days after inhibition of MMP-9 and MMP-12. As a comparison, Melatonin only had a marginal effect. (D) AZD3342 significantly suppresses MMP-9 and MMP-12 activity. (E) AZD3342 significantly suppressed the expression of proinflammatory pain cytokines, with Melatonin having a small effect. (F) Representative Spike 2 processed CAP traces at 6 weeks after treatment with AZD3342 and Melatonin. AZD3342 restored a significant CAP wave after DC injury. (G) AZD3342 improved ladder crossing performance (locomotor function) over 6 weeks. (H) AZD3342 improved tape sensing and removal performance times (sensory function) over 6 weeks compared to vehicle or melatonin treated rats. Data are expressed as means ± SEM. *n* = 6 mice/group, 2 independent experiments, total *n* = 12 mice/group. *** = P=0.0001, one-way ANOVA with Dunnett’s post hoc test. # = P<0.0011, linear mixed models; ## = P<0.0011, generalized linear mixed models. NOTE: AZD1236 treatment was provided immediately after injury.


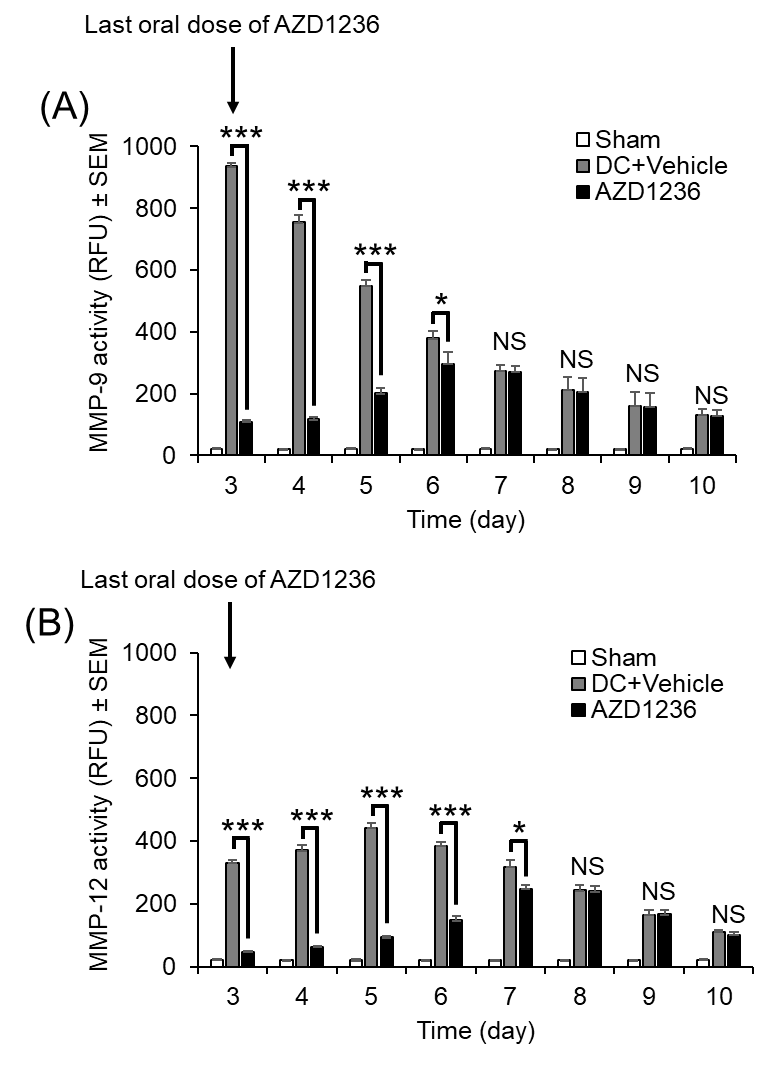


**FIGURE S11.** **Return of MMP-9 and MMP-12 activity at the lesion site to normal DC-injury-induced levels by 4-5 days after the last oral dose of AZD1236 in mice.** (**A**) MMP-9 acvitiy returns to injury-induced levels by day 7, taking 4 days to retrun to these levels after withdrawal of AZD1236. (**B**) MMP-12 acvitiy returns to injury-induced levels by day 8, taking 5 days to return to these after after withdrawal of AZD1236. * = *P*<0.05; *** = *P*=0.0001, one-way ANOVA with Dunnett’s post hoc test. *n* = 6 mice/group, 2 independent repeats (total *n* = 12 mice/group). NOTE: AZD1236 treatment was provided immediately after injury.

**SUPPLEMENTARY TABLES**

**TABLE S1. MMP inhibitor concentrations used in this study.** Unless referenced, all doses have been tested in this study. IP = intraperitoneal, It = intrathecal, SC = subcutaneous. Sur1-regulated NCCa-ATP = sulfonylurea receptor 1-regulated NC_Ca_-ATP channels.

| **Inhibitor** | **Source** | **Catalogue**  **no.** | **Activity** | **Doses tested**  **mg/kg** | **Route and Frequency** |
| --- | --- | --- | --- | --- | --- |
| AZD1236 | AstraZeneca, Cambridge, UK | N/A | MMP-9 and -12 | 100, 200 and 300  2.5, 5 and 10 | Oral, twice daily  It, twice daily |
| AZD3342 | AstraZeneca, Cambridge, UK | N/A | MMP-9 and -12 | 15, 75 and 375 | Oral, twice daily |
| GM6001 | Tocris, Oxford, UK | 2983/10 | MMP-1, -2, -3, -8 and -9 | 100-500 | IP, twice daily ^29^ |
| SB-3CT | Tocris, Oxford, UK | 6088/10 | MMP-2 and -9 | 10, 20, 25, 30, 35 | IP at 2 and 4 hours after injury then once daily ^30^ |
| MMP-9 Inhibitor I | Merck, Watford, UK | 444278 | MMP-9 | 100-500 | IP, twice daily |
| MMP408 | Merck, Watford, UK | 444291 | MMP-12 | 100, 200 and 300 | IP, twice daily |
| ND-378 | MedKoo, Mirrisville, NC, USA | 526432 | MMP-2 | 15, 25, 35, 50, 60 | IP at 24 hours, SC 1 hour after first IP dose, then SC doses once daily ^31^ |
| Melatonin | Tocris, Oxford, UK | 3550/50 | Aquaporin-4 | 50-400 | IP, twice daily ^32^ |
| Minocycline | Tocris, Oxford, UK | 3268/50 | No effect on MMP activity | 50 | 50mg for 2 days then 25mg/kg ^33^ |
| Riluzole | Tocris, Oxford, UK | 0768/25 | GABA uptake inhibitor | 8 | IP, 12 hours pre-SCI and twice daily for 3 days ^34^ |
| Glibenclamide | Tocris, Oxford, UK | 0911/100 | Sur1-regulated NCCa-ATP | 0.01 | IP, loading dose of 0.01mg/kg then 200ng/hour for 7 days ^35^. |

REFERENCES

1. Almutiri S, Berry M, Logan A, Ahmed Z. Non-viral-mediated suppression of AMIGO3 promotes disinhibited NT3-mediated regeneration of spinal cord dorsal column axons. *Sci Rep.* 2018;8(1):10707.

2. Stevens AR, Ahmed U, Vigneswara V, Ahmed Z. Pigment Epithelium-Derived Factor Promotes Axon Regeneration and Functional Recovery After Spinal Cord Injury. *Mol Neurobiol.* 2019;56(11):7490-7507.

3. Farrukh F, Davies E, Berry M, Logan A, Ahmed Z. BMP4/Smad1 Signalling Promotes Spinal Dorsal Column Axon Regeneration and Functional Recovery After Injury. *Mol Neurobiol.* 2019;56(10):6807-6819.

4. Tuxworth RIT, M.J.; Anduaga, A-M.; Hussien-Ali, A.; Chatzimatthaiou, S.; Longland, J.; Thompson, A.M.; Almutiri, S.; Alifragis, P.; Kyriacou, C.P.; Kysela, B.; Ahmed, Z. Attenuating the DNA damage response to double-strand breaks restores function in models of CNS neurodegeneration. *Brain Communications.* 2019;1(1):fcz005.

5. Kitchen P, Salman MM, Halsey AM, et al. Targeting Aquaporin-4 Subcellular Localization to Treat Central Nervous System Edema. *Cell.* 2020;181(4):784-799 e719.

6. Ahmed Z, Bansal D, Tizzard K, et al. Decorin blocks scarring and cystic cavitation in acute and induces scar dissolution in chronic spinal cord wounds. *Neurobiol Dis.* 2014;64:163-176.

7. Surey S, Berry M, Logan A, Bicknell R, Ahmed Z. Differential cavitation, angiogenesis and wound-healing responses in injured mouse and rat spinal cords. *Neuroscience.* 2014;275:62-80.

8. Oladosu FA, Ciszek BP, O'Buckley SC, Nackley AG. Novel intrathecal and subcutaneous catheter delivery systems in the mouse. *J Neurosci Methods.* 2016;264:119-128.

9. Read ML, Mir S, Spice R, et al. Profiling RNA interference (RNAi)-mediated toxicity in neural cultures for effective short interfering RNA design. *J Gene Med.* 2009;11(6):523-534.

10. Rivlin AS, Tator CH. Effect of duration of acute spinal cord compression in a new acute cord injury model in the rat. *Surg Neurol.* 1978;10(1):38-43.

11. Esposito E, Paterniti I, Mazzon E, et al. MK801 attenuates secondary injury in a mouse experimental compression model of spinal cord trauma. *BMC Neurosci.* 2011;12:31.

12. Li S, Tator CH. Effects of MK801 on evoked potentials, spinal cord blood flow and cord edema in acute spinal cord injury in rats. *Spinal Cord.* 1999;37(12):820-832.

13. Ahmed Z, Dent RG, Leadbeater WE, Smith C, Berry M, Logan A. Matrix metalloproteases: degradation of the inhibitory environment of the transected optic nerve and the scar by regenerating axons. *Mol Cell Neurosci.* 2005;28(1):64-78.

14. Li YQ, Ballinger JR, Nordal RA, Su ZF, Wong CS. Hypoxia in radiation-induced blood-spinal cord barrier breakdown. *Cancer Res.* 2001;61(8):3348-3354.

15. Nag S. Blood-brain barrier permeability using tracers and immunohistochemistry. *Methods Mol Med.* 2003;89:133-144.

16. Zea-Aragon Z, Terada N, Ohno N, Fujii Y, Baba T, Ohno S. Effects of anoxia on serum immunoglobulin and albumin leakage through blood-brain barrier in mouse cerebellum as revealed by cryotechniques. *J Neurosci Methods.* 2004;138(1-2):89-95.

17. Gordh T, Chu H, Sharma HS. Spinal nerve lesion alters blood-spinal cord barrier function and activates astrocytes in the rat. *Pain.* 2006;124(1-2):211-221.

18. Asher RA, Morgenstern DA, Fidler PS, et al. Neurocan is upregulated in injured brain and in cytokine-treated astrocytes. *J Neurosci.* 2000;20(7):2427-2438.

19. Tang X, Davies JE, Davies SJ. Changes in distribution, cell associations, and protein expression levels of NG2, neurocan, phosphacan, brevican, versican V2, and tenascin-C during acute to chronic maturation of spinal cord scar tissue. *J Neurosci Res.* 2003;71(3):427-444.

20. Wang Y, Gao Z, Zhang Y, et al. Attenuated Reactive Gliosis and Enhanced Functional Recovery Following Spinal Cord Injury in Null Mutant Mice of Platelet-Activating Factor Receptor. *Mol Neurobiol.* 2016;53(5):3448-3461.

21. Moussaud S, Draheim HJ. A new method to isolate microglia from adult mice and culture them for an extended period of time. *J Neurosci Methods.* 2010;187(2):243-253.

22. McIntyre C, Saville J, Fuller M. Collection of cerebrospinal fluid from murine lateral ventricles for biomarker determination in mucopolysaccharidosis type IIIA. *J Neurosci Methods.* 2019;324:108314.

23. Rosas M, Davies LC, Giles PJ, et al. The transcription factor Gata6 links tissue macrophage phenotype and proliferative renewal. *Science.* 2014;344(6184):645-648.

24. Green TD, Park J, Yin Q, et al. Directed migration of mouse macrophages in vitro involves myristoylated alanine-rich C-kinase substrate (MARCKS) protein. *J Leukoc Biol.* 2012;92(3):633-639.

25. Hains BC, Saab CY, Lo AC, Waxman SG. Sodium channel blockade with phenytoin protects spinal cord axons, enhances axonal conduction, and improves functional motor recovery after contusion SCI. *Exp Neurol.* 2004;188(2):365-377.

26. Farrukh F, Davies E, Berry M, Logan A, Ahmed Z. BMP/Smad1 signalling promotes spinal dorsal column axon regeneration and functional recovery after injury. *Mol Neurobiol.* 2019.

27. Fagoe ND, Attwell CL, Eggers R, et al. Evaluation of Five Tests for Sensitivity to Functional Deficits following Cervical or Thoracic Dorsal Column Transection in the Rat. *PLoS One.* 2016;11(3):e0150141.

28. Nasirinezhad F, Gajavelli S, Priddy B, Jergova S, Zadina J, Sagen J. Viral vectors encoding endomorphins and serine histogranin attenuate neuropathic pain symptoms after spinal cord injury in rats. *Mol Pain.* 2015;11:2.

29. Noble LJ, Donovan F, Igarashi T, Goussev S, Werb Z. Matrix metalloproteinases limit functional recovery after spinal cord injury by modulation of early vascular events. *J Neurosci.* 2002;22(17):7526-7535.

30. Cui J, Chen S, Zhang C, et al. Inhibition of MMP-9 by a selective gelatinase inhibitor protects neurovasculature from embolic focal cerebral ischemia. *Mol Neurodegener.* 2012;7:21.

31. Gao M, Zhang H, Trivedi A, et al. Selective Inhibition of MMP-2 Does Not Alter Neurological Recovery after Spinal Cord Injury. *ACS Chem Neurosci.* 2016;7(11):1482-1487.

32. Li C, Chen X, Qiao S, et al. Melatonin lowers edema after spinal cord injury. *Neural Regen Res.* 2014;9(24):2205-2210.

33. Wells JE, Hurlbert RJ, Fehlings MG, Yong VW. Neuroprotection by minocycline facilitates significant recovery from spinal cord injury in mice. *Brain.* 2003;126(Pt 7):1628-1637.

34. Caglar YS, Demirel A, Dogan I, et al. Effect of Riluzole on Spinal Cord Regeneration with Hemisection Method Before Injury. *World Neurosurg.* 2018;114:e247-e253.

35. Simard JM, Tsymbalyuk O, Keledjian K, Ivanov A, Ivanova S, Gerzanich V. Comparative effects of glibenclamide and riluzole in a rat model of severe cervical spinal cord injury. *Exp Neurol.* 2012;233(1):566-574.
